# Spectroscopy and crystallography define carotenoid oxygenases as a new subclass of mononuclear non-heme Fe^II^ enzymes

**DOI:** 10.1016/j.jbc.2025.108444

**Published:** 2025-03-25

**Authors:** Dory E. DeWeese, Michael P. Everett, Jeffrey T. Babicz, Anahita Daruwalla, Edward I. Solomon, Philip D. Kiser

**Affiliations:** 1 Department of Chemistry, Stanford University, Stanford, California, USA; 2 Department of Physiology & Biophysics, University of California, Irvine School of Medicine, Irvine, California, USA; 3 SLAC National Accelerator Laboratory, Stanford University, Menlo Park, California, USA; 4 Research Service, VA Long Beach Healthcare System, Long Beach, California, USA

**Keywords:** carotenoid, dioxygenase, iron, isoeugenol, nitric oxide, piceatannol, stilbene

## Abstract

Carotenoid cleavage dioxygenases (CCDs) are non-heme Fe^II^ enzymes that catalyze the oxidative cleavage of alkene bonds in carotenoids, stilbenoids, and related compounds. How these enzymes control the reaction of dioxygen (O_2_) with their alkene substrates is unclear. Here, we apply spectroscopy in conjunction with X-ray crystallography to define the iron coordination geometry of a model CCD, CAO1 (*Neurospora crassa* carotenoid oxygenase 1), in its resting state and following substrate binding and coordination sphere substitutions. Resting CAO1 exhibits a five-coordinate (5C), square pyramidal Fe^II^ center that undergoes steric distortion toward a trigonal bipyramidal geometry in the presence of piceatannol. Titrations with the O_2_-analog, nitric oxide, show a >100-fold increase in iron–nitric oxide affinity upon substrate binding, defining a crucial role for the substrate in activating the Fe^II^ site for O_2_ reactivity. The importance of the 5C Fe^II^ structure for reactivity was probed through mutagenesis of the second-sphere Thr151 residue of CAO1, which occludes ligand binding at the sixth coordination position. A T151G substitution resulted in the conversion of the iron center to a six-coordinate state and a 135-fold reduction in apparent catalytic efficiency toward piceatannol compared with the wildtype enzyme. Substrate complexation resulted in partial six-coordinate to 5C conversion, indicating solvent dissociation from the iron center. Additional substitutions at this site demonstrated a general functional importance of the occluding residue within the CCD superfamily. Taken together, these data suggest an ordered mechanism of CCD catalysis occurring *via* substrate-promoted solvent replacement by O_2_. CCDs thus represent a new class of mononuclear non-heme Fe^II^ enzymes.

Carotenoid cleavage dioxygenases (CCDs) are a broadly distributed superfamily of enzymes that catalyze the reaction of dioxygen (O_2_) with carotenoids, stilbenoids, and related redox-active polyenes (Fig. 1*A*) (1). These enzymes use a mononuclear non-heme Fe^II^ center to activate O_2_ for reaction with a specific alkene group in the substrate, generating carbonyl-containing products of importance in biological processes such as hormonal signaling and light detection (2, 3, 4).

**Figure 1:**
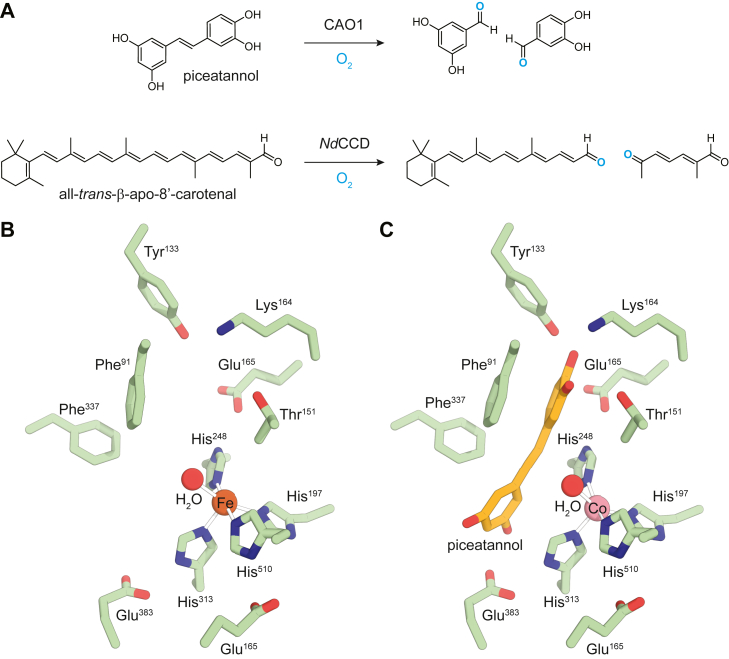
**Activity and structure of carotenoid-cleaving enzymes.***A*, examples of alkene bond cleavage activity within the CCD superfamily. *B*, crystal structure of the resting Fe^II^–CAO1 (Protein Data Bank accession code: 5U8X) (9). *C*, crystal structure of piceatannol-bound Co^II^-CAO1 (Protein Data Bank accession code: 5U97) (9). CAO1, *Neurospora crassa* carotenoid oxygenase 1; CCD, carotenoid cleavage dioxygenase.

CCDs belong to the broad group of oxygen-activating, organic cofactor–independent, mononuclear non-heme Fe^II^ dioxygenases (Table S1, non–cofactor dependent) (5, 6). Other members of this class include the thiol dioxygenases, the extradiol dioxygenases, and the Rieske dioxygenases (6). While CCDs do not require an organic cofactor, they are notably distinct from other members of the cofactor-independent class. Unlike many mononuclear non-heme iron dioxygenases that coordinate their Fe^II^ centers using a 2-His, 1-Glu/Asp “facial triad” motif (7), CCDs use a 4-His cluster, three of which hydrogen bond to second-sphere carboxylates (Glu/Asp) (Fig. 1*B*) (2). This arrangement leaves two *cis* open positions on the iron potentially available for ligand binding. While one coordination position is occupied by a solvent ligand as demonstrated by several crystallographic and spectroscopic investigations (2, 5, 8, 9, 10, 11), the other is vacant because of apparent occlusion by a nearby methyl group of a semiconserved, second sphere residue (*i.e.*, Thr/Val/Ile, Fig. S1), making CCDs five-coordinate (5C) in their resting Fe^II^ states. Further characterization has demonstrated that the substrate does not directly coordinate the metal center, and the iron center apparently remains 5C in the presence of substrate (Fig. 1*C*) (9, 12), which contrasts with the other members of the non–cofactor-dependent class. In the cysteine oxidase/dioxygenases and the extradiol dioxygenases, the resting iron sites are mostly six-coordinate (6C), and the direct coordination of substrate to the Fe^II^ site lowers its reduction potential forming a 5C site that is competent to react with O_2_ (13, 14, 15). In the Rieske dioxygenases, where the Rieske center serves as an additional electron donor, the catalytic resting Fe^II^ site is 6C and stable in O_2_ (16). While the substrate does not directly bind to the iron, it induces loss of a solvent ligand to form a 5C site for reaction with O_2_ (16). The cofactor-dependent non-heme iron enzymes (Table S1) parallel the Rieske dioxygenases in that substrate binding opens a coordination position on the Fe^II^ for O_2_ activation but only in the presence of the two-electron-donating organic cofactor.

These differences in the resting and substrate-bound structures of the CCDs as compared with the other mononuclear non-heme iron enzymes suggest that CCDs may use an alternative mechanism for enabling O_2_ reactivity at the iron center. Computational studies have proposed several possible mechanisms for the oxidative cleavage of stilbenoids/carotenoids by CCDs (17, 18, 19, 20). Two possible mechanisms for the initial reaction have been suggested, where O_2_ binds to the Fe^II^ center either with or without solvent dissociation (17, 20). The initial Fe–O_2_ intermediate is calculated to form concurrent with substrate oxidation, resulting in an Fe^II^-superoxide and substrate radical. Reaction of the Fe^II^-superoxide with the substrate radical then results in either a dioxetane or an epoxide intermediate, with further transformations leading to the aldehyde–ketone cleavage products (17, 18, 19, 20). While these calculations have been illuminating, consideration of the initial reaction of the Fe^II^ site with O_2_ and the impact of the occluding residue and the organic substrate on this reaction have been limited. Some crystallographic studies have suggested that CCD iron–oxygen adducts can form in the absence of the organic substrate (21, 22), although these interpretations are controversial (23).

In this study, we examined the impact of substrate binding and substitutions of the second-sphere occluding residue on the iron coordination of the model stilbenoid-cleaving CCD, CAO1 (*Neurospora crassa* carotenoid oxygenase 1) (24), and the apocarotenoid-cleaving CCD, *Nd*CCD (*Nitrosotalea devanaterra* CCD) (8), using near-infrared (NIR), low temperature (LT) magnetic CD (MCD) spectroscopy, and X-ray crystallography. Together with supporting CD spectroscopy, UV–visible absorption spectroscopy, and kinetic studies, this study elucidates the role of the occluding residue in determining the Fe^II^ site structure, the importance of the 5C resting state for efficient catalytic function, and the role of the substrate in preparing the Fe^II^ center for reaction with O_2_.

## Results

### MCD definition of the wildtype CAO1 resting-state iron center structure and correlation to crystallography

**Figure 2:**
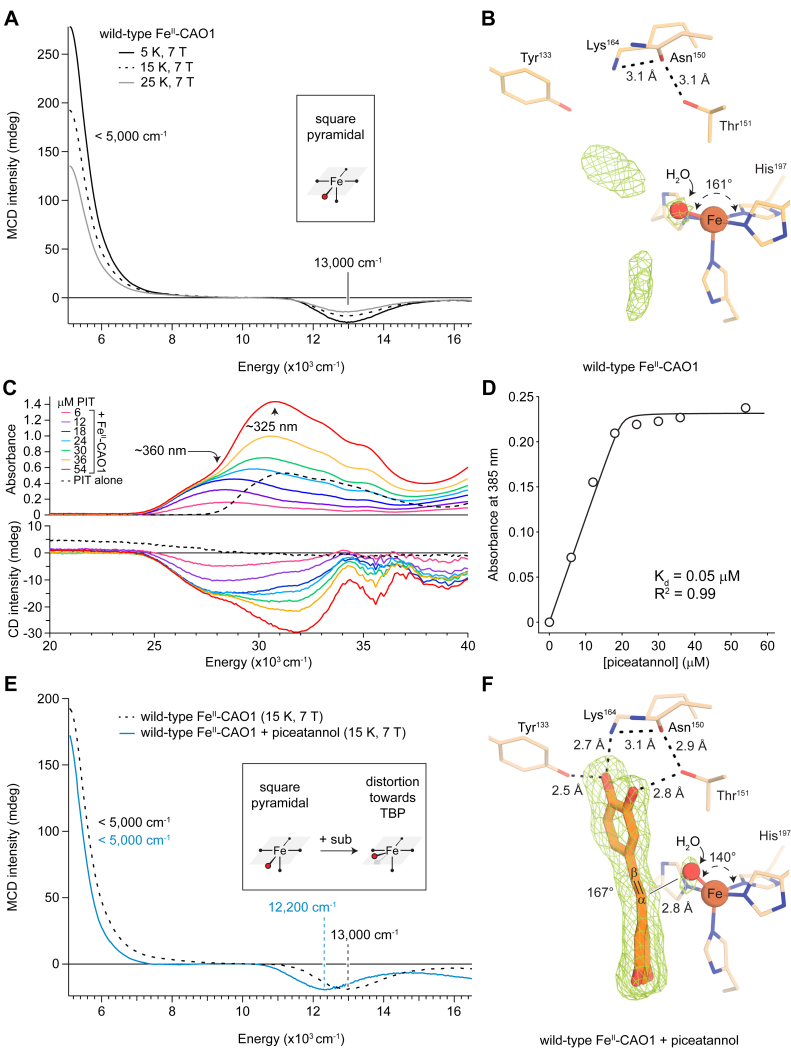
**MCD–CD spectroscopic and crystallographic definition of the CAO1 Fe^II^ center in the absence and presence of piceatannol.***A*, LT MCD spectrum of resting wildtype Fe^II^–CAO1. *B*, crystal structure of resting wildtype Fe^II^–CAO1. *C*, wildtype Fe^II^–CAO1 titrated with increasing equivalents of piceatannol and monitored by UV–visible absorption (*top*) and CD (*bottom*) spectroscopies. *D*, least-squares fitting of the saturable site absorbance data to the quadratic binding equation. *E*, LT MCD spectrum of resting (*black dashed line*) and substrate-bound (*blue*) wildtype Fe^II^–CAO1. The spectral shift in the presence of substrate is consistent with the Fe^II^ site undergoing a trigonal bipyramidal (TBP) distortion. *F*, crystal structure of piceatannol-bound wildtype Fe^II^–CAO1. The *green mesh* represents NCS-averaged, sigma A-weighted *F*_o_–*F*_c_ omit electron density contoured at the 5.5 RMSD level. *Dashed straight lines* indicate hydrogen bonding interactions. The His197N^ε^–Fe–H_2_O angles and piceatannol Cα–Cβ torsional angle are labeled. The data in *A*, *C*, and *E* are representative of two independent experiments. CAO1, *Neurospora crassa* carotenoid oxygenase 1; LT, low temperature; MCD, magnetic CD.

The NIR LT MCD spectrum of anaerobic, wildtype Fe^II^–CAO1 features two ligand-field (LF) transitions, an intense positive feature at <5000 cm^-1^ and a weaker negative feature at 13,000 cm^-1^ (Fig. 2*A*). The energies of these features are consistent with a 5C distorted square pyramidal Fe^II^ center (25). The two LF transitions gain MCD intensity with decreasing temperature (Fig. 2*A*) and increasing field strength (Fig. S2), consistent with the transitions originating from a paramagnetic ground state (26). The distorted square pyramidal iron structure derived from MCD spectroscopy agrees well with the wildtype Fe–CAO1 crystallographic structure (Fig. 2*B* and Table S2), which shows a 5C iron center containing a single coordinated solvent molecule together with the four His ligands arranged with a distorted square pyramidal geometry (9).

To estimate the protonation state of the coordinated solvent molecule (water *versus* hydroxide), MCD spectra were obtained from samples prepared at pH values of 6.3, 7.2, 8.9, and 9.5 (Fig. S3). The samples at pH 6.3 and 7.2 are similar with two features at <5000 and 13,000 cm^-1^. Upon increasing the pH to 8.9, there is growth of two weak features at 9100 and ∼11,200 cm^-1^ with a resulting shift in the negative, high-energy band to 13,200 cm^-1^. Increasing the pH to 9.5 resulted in little change to the MCD spectrum. The normalized MCD intensity at 9000 cm^-1^ *versus* pH was fit to the Henderson–Hasselbach equation giving a p*K*_a_ of 8.2, which may represent an approximate p*K*a of the bound water (Fig. S3). This estimate indicates that at biologically relevant pH values, the bound solvent is water rather than hydroxide, consistent with a previous X-ray absorption spectroscopy study (12) and similar to the 3-His Fe^II^–water site in DKE1, which has a p*K*_a_ of ∼8.2 (27). The ∼2.4 Å iron-solvent bond length derived from crystallography (Table S3) is also more consistent with water than hydroxide, as Fe^II^-hydroxo coordinate bonds are typically ∼2.2 Å in length (28, 29).

### CD–MCD spectroscopic and crystallographic characterization of the wildtype CAO1–substrate complex

Next, the binding of the CAO1 substrate piceatannol (9, 24) to wildtype Fe^II^–CAO1 was measured by CD and UV–visible absorption spectroscopy. Titration of piceatannol into anerobic solutions of CAO1 produced the spectra shown in Figure 2*C*. At low substrate concentrations, the spectra showed an absorbance maximum at ∼360 nm that progressively saturates. At higher concentrations, an absorbance maximum at ∼325 nm, with an absorbance profile like that of the free piceatannol substrate (*dashed black line*), becomes prominent and does not saturate. Simultaneous measurement of CD spectra for these samples showed that both the 360 and 325 nm species are optically active, unlike the behavior observed for free piceatannol, which is achiral. The optical activity for the ∼325 nm species originates from piceatannol bound to CAO1 at non–active site regions, as observed by crystallography (Fig. S4). Based on these saturation behaviors and by comparison to free substrate, we assign the 360 and 325 nm absorbance peaks to active site–bound and non–active site–bound piceatannol, respectively. The binding of piceatannol to the CAO1 active site induces a pronounced bathochromic shift in the piceatannol absorbance spectrum. Least-squares fitting of the absorbance data for the saturable feature to a one-site quadratic binding equation resulted in a dissociation constant (*K*_*d*_) of 0.05 ± 0.16 μM for piceatannol binding to the wildtype Fe^II^–CAO1 active site (Fig. 2*D*). Consequentially, an equimolar mixture of Fe^II^–CAO1 and piceatannol at the high micromolar to millimolar concentrations used in subsequent experiments is assured to result in a stoichiometric enzyme–substrate (ES) complex.

The NIR LT MCD spectrum of anaerobic, piceatannol-bound Fe^II^–CAO1 (Fig. 2*E*, *blue*) exhibits two LF transitions, an intense positive feature at <5000 cm^-1^ and a weak negative feature at 12,200 cm^-1^, indicating that the iron site remains 5C in the presence of substrate. The shift of the high-energy band from 13,000 cm^-1^ in the resting site to 12,200 cm^-1^ in the substrate-bound form indicates a distortion toward a more trigonal bipyramidal geometry when substrate binds to the active site (25). This structural change was corroborated by the substrate-bound wildtype Fe^II^–CAO1 crystallographic structure (Table S2), which showed a distortion from a square pyramidal toward trigonal bipyramidal geometry because of substrate-induced movement of the solvent molecule out of the equatorial plane toward the axial position. This is shown in Figure 2*F*, with ∠His197N^ε^–Fe–H_2_O decreasing from 161° in the resting enzyme to 140° in the substrate-bound form (Table S3), which is attributed to a steric influence of the bound substrate. Other notable features of the active site piceatannol molecule include a strong hydrogen bonding interaction (∼2.5 Å) of its 4′-hydroxyl moiety with Tyr133 as well as a nonplanar dihedral angle of the interphenyl scissile alkene bond (167°), which compare favorably to previously determined structures of Co-substituted CCDs in complex with stilbenoid substrates (9, 10).

### Impact of substrate on nitric oxide binding to Fe^II^–CAO1

**Figure 3:**
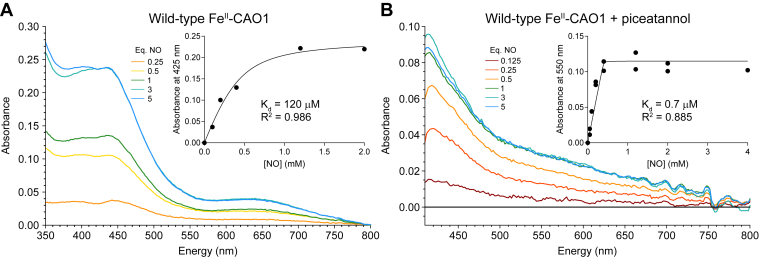
**Impact of piceatannol on the CAO1 iron center affinity for nitric oxide (NO).***A*, UV–visible absorption spectra of resting Fe^II^–CAO1 binding NO in a series of increasing NO equivalents. *B*, UV–visible absorption spectra of piceatannol-bound Fe^II^–CAO1 binding NO in a series of increasing equivalents of NO. *Insets* show least-squares fits of the quadratic binding equation to the titration data. The data are representative of two independent experiments. CAO1, *Neurospora crassa* carotenoid oxygenase 1.

There are conflicting data regarding the O_2_-binding ability of the resting Fe^II^ site in stilbene-cleaving CCDs. Some studies indicate that the resting Fe^II^ site is inert to O_2_ (9), whereas others suggest that the Fe^II^ site can react with O_2_ even in the absence of substrate (21). To lend insight into the role organic substrate plays in O_2_ binding, nitric oxide (NO) was used as an analog for O_2_ binding, where NO binding behavior generally parallels O_2_, but the resultant Fe^III^–NO^-^ complex is unreactive toward substrate (30, 31). The binding of NO to Fe^II^–CAO1 in resting and substrate-bound states was measured using UV–visible absorption spectroscopy. Figure 3*A* shows an NO titration for the resting enzyme, where the absorption saturates at three added equivalents of NO (*aqua trace*). Using the absorption intensity at 425 nm, the *K*_*d*_ for NO binding is 120 μM (95% upper confidence limit = 320 μM) (Fig. 3*A*, *inset*). Relative to the resting site, the substrate-bound Fe^II^ site shows an increased affinity for NO with the absorption saturating near one equivalent of NO (*green trace*). The *K*_*d*_ for NO binding to the substrate-bound active site obtained by fitting the absorption intensity at 550 nm is 0.7 μM (95% upper confidence limit = 9.4 μM) (Fig. 3*B*, *inset*). Therefore, in the presence of substrate, the binding affinity of NO increases by more than two orders of magnitude, which equates to a >3 kcal/mol more favorable ΔΔG° for NO binding in the presence of the organic substrate.

### Spectroscopic and structural properties of T151G-substituted CAO1

The unusual 5C resting state structure observed for CAO1 is enforced by an occluding Thr side-chain proximate to the open Fe^II^ coordination position, as demonstrated by the solvent-excluded surface of Fe^II^–CAO1 (Fig. S5*A*). To investigate whether reduction in side-chain volume would allow solvent binding at the sixth position, site-directed mutagenesis was used to introduce a T151G substitution in CAO1, thereby opening the maximal potential space for ligand coordination.

**Figure 4:**
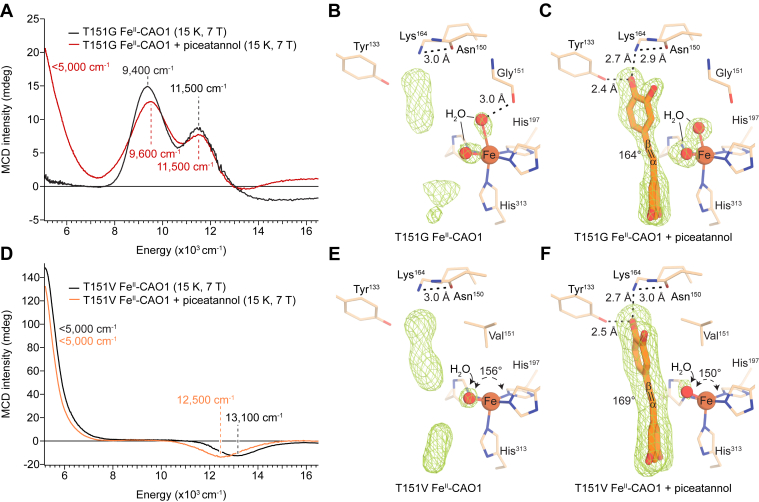
**Impact of occluding site substitutions on the CAO1 iron center structure in the absence and presence of piceatannol.***A*, MCD spectra of resting (*black*) and substrate-bound (*red*) T151G Fe^II^–CAO1. The spectra are consistent with a 6C, distorted octahedral resting-state Fe^II^ site that converts to a 5C–6C mixture in the presence of piceatannol. *B*, crystal structure of resting T151G Fe^II^–CAO1. *C*, crystal structure of piceatannol-bound T151G Fe^II^–CAO1. *D*, MCD spectra of resting (*black*) and substrate-bound (*orange*) T151V Fe^II^–CAO1. The spectra are consistent with a 5C square pyramidal resting state Fe^II^ structure in the resting state that undergoes a trigonal bipyramidal distortion in the presence of piceatannol. *E*, crystal structure of resting T151V Fe^II^–CAO1. *F*, crystal structure of piceatannol-bound T151V Fe^II^–CAO1. The *green mesh* represents sigma A-weighted *F*_o_–*F*_c_ omit electron density contoured at the 4.2 (*B*), 5.5 (*C*), 5.5 (*E*), and 3.6 (*F*) RMSD levels. The omit density was NCS-averaged except in (*B*) where density for chain B is shown on account of solvent positional heterogeneity between the different chains of the asymmetric unit. *Dashed straight lines* indicate hydrogen bonding interactions. The solvent–iron–His197 angles and piceatannol Cα–Cβ torsional angles are labeled. The data in *A* and *D* are representative of two independent experiments. 5C, five-coordinate; 6C, six-coordinate; CAO1, *Neurospora crassa* carotenoid oxygenase 1; MCD, magnetic CD.

The NIR LT MCD spectrum of anaerobic T151G Fe^II^–CAO1 (*black*) exhibits two LF transitions of moderate intensity at 9400 and 11,500 cm^-1^ (Fig. 4*A*). The energies of these features are consistent with a 6C distorted octahedral Fe^II^ center (25), a marked change in structure from the 5C square pyramidal site found for resting wildtype Fe^II^–CAO1 (Fig. 2, *A* and *B*). The coordination environment of T151G Fe^II^–CAO1 was also investigated crystallographically (Fig. 4*B*). Globally, the structure of T151G Fe^II^–CAO1 closely resembled the wildtype protein with an overall Cα RMSD of 0.125 Å. Two discrete density map peaks were observed in proximity to the iron center indicating two coordinated solvent molecules. One of these (solvent 1) corresponded to the normally bound water molecule located *trans* to His197. The second peak, located across from His313, was also satisfactorily modeled with a water molecule (solvent 2). The newly accessible space for solvent coordination was readily apparent in the solvent-excluded surface of the T151G Fe^II^–CAO1 variant (Fig. S5*B*). The average Fe–O bond lengths for solvents 1 and 2 were 2.52 ± 0.15 Å and 2.80 ± 0.10 Å, respectively, indicating relatively weak coordination (Table S3). These bond lengths are somewhat longer than the ∼2.4 Å Fe^II^–solvent interaction observed for the wildtype protein. The ∠His197N^ε^–Fe–H_2_O for solvent 1 is 170°, whereas solvent 2 is distorted away from a *trans* geometry with an ∠His313N^ε^–Fe–H_2_O of ∼153°. This distortion arises from the solvent avoiding a steric clash with a nearby Ala196 side chain as well as its engagement in a weak hydrogen bonding interaction with the main chain carbonyl oxygen of residue 151. As in the wildtype Fe^II^–CAO1 structure (Fig. 2*B*), there were two additional residual density peaks in the active-site pocket that may represent diffusely associated water molecules (Fig. 4*B*, *green mesh*).

The effect of substrate binding on the Fe^II^ site of T151G CAO1 was determined by NIR LT MCD spectroscopy. The spectrum of the substrate-bound Fe^II^ site (Fig. 4*A*, *red*) shows three bands at <5000, 9600, and 11,500 cm^-1^. The presence of a low energy feature at <5000 cm^-1^ indicates the formation of a 5C species upon substrate binding. The second 5C LF transition overlaps with the feature at 9600 cm^-1^, as evidenced by growth of the shoulder at ∼8000 cm^-1^ in the substrate-bound complex. The spectrum was adequately fit by four bands with maxima at 4700, 8350, 9570, and 11,500 cm^-1^ (Fig. S6*A*). Fits that included only three transitions necessitated an unrealistic full-width at half-maximum value for the transition at ∼9500 cm^-1^ (Fig. S6*B*). The energies and intensity patterns of these four transitions are consistent with one 6C and one 5C species, constituting ∼85% and ∼15% of the sample, respectively, as estimated by comparison of the intensities at 11,500 cm^-1^. These data indicate that substrate binding leads to dissociation of one iron-bound water in a subset of the molecules in the sample (25).

Crystallography on the T151G Fe^II^–CAO1 piceatannol complex showed a piceatannol binding conformation in T151G Fe^II^–CAO1 resembling that observed for the wildtype enzyme, the main difference being a ∼10° rotation of the β-ring toward the vacancy left from the absent Thr side chain (Table S2 and Fig. 4*C*). Two solvent molecules remained bound to the iron center *trans* to His197 and His313 (Fig. 4*C*). Notably the iron-solvent bond lengths were shortened by ∼0.4 to 0.5 Å in the ES complex as compared with the resting T151G Fe^II^–CAO1 structure (Table S3). However, the resolution of the diffraction data was insufficient to allow modeling of the mixture of 5C and 6C species found by MCD spectroscopy.

The ligand-binding properties of the T151G CAO1 ES complex were further investigated through NO titration studies (Fig. S7). The UV–visible absorbance data exhibited a biphasic appearance suggesting a mixture of two different species in the sample (Fig. S7*A*). Indeed, a two-species binding model with a higher affinity (*K*_*d*_ = 3 μM) site forming 24% of the sample and a lower affinity (*K*_*d*_ = 13 μM) site making up 76% of the sample provided a superior fit to the data compared with a one-species model (Fig. S7, *B* and *C*). Attempts to model the wildtype Fe^II^–CAO1 ES–NO data with the two-species model resulted in unrealistic *K*_*d*_ values. The species percentages reasonably match the proportion of 5C and 6C species observed for the T151G Fe^II^–CAO1 ES complex by MCD spectroscopy, suggesting that the higher affinity NO-binding species corresponds to the 5C iron site.

### Spectroscopic and structural properties of T151V-substituted CAO1

To further investigate the contribution of the occluding residue to the CAO1 active site structure, a T151V substitution was introduced at the occluding site, as found in some carotenoid-cleaving CCDs like *Nd*CCD (Fig. S1) (8). The NIR LT MCD spectrum of anaerobic T151V Fe^II^–CAO1 (Fig. 4*D*, *black*) exhibits two LF transitions, an intense positive feature at <5000 cm^-1^ and a weaker negative feature at 13,100 cm^-1^, consistent with a 5C distorted square pyramidal Fe^II^ center (25). Comparing the resting T151V Fe^II^–CAO1 spectrum (Fig. 4*D*, *black*) to that of the wildtype Fe^II^–CAO1 spectrum (Fig. 2*A*, *black*) shows that the T151V substitution minimally perturbs the active site structure, given by the small, 100 cm^-1^ upshift in the negative, higher energy band. The similarity in structure between wildtype and T151V Fe^II^–CAO1 was confirmed by crystallography (Table S2) as shown in Figure 4*E* with a pairwise Cα RMSD of 0.1 Å between the two structures. The substituted Val side retains the ability to occlude the sixth coordination position as shown by the solvent-excluded surface (Fig. S5*C*), consistent with the MCD data. Although the Val151 side-chain conformation resembled that of the wildtype Thr residue, it was shifted toward the active-site cavity by ∼0.2 to 0.4 Å and was also closer to the iron center by ∼0.2 Å. The loss of hydrogen bonding capacity associated with the T151V substitution led to shifts in the Asn150 side chain and a nearby water molecule away from the methyl group of Val that had replaced the corresponding Thr hydroxyl group in wildtype Fe^II^–CAO1. Also notable in the T151V mutant was a shift in the positioning of the iron by ∼0.4 Å toward His197. However, the distorted square pyramidal structure of the T151V Fe^II^ center closely resembled that of wildtype Fe^II^–CAO1 with iron–His bond lengths of 2.1 to 2.2 Å and an iron-solvent bond length of ∼2.4 Å (Table S3).

To characterize the effect of substrate binding on the Fe^II^ center, NIR LT MCD was measured on the piceatannol-bound form of anaerobic T151V Fe^II^–CAO1 (Fig. 4*D*, *orange*). The spectrum shows two LF transitions at <5000 and 12,500 cm^-1^, consistent with a 5C square pyramidal structure (25). Similarly to wildtype Fe^II^–CAO1, substrate binding to the active site induced a shift in the high energy feature from 13,100 cm^-1^ in the resting enzyme to 12,500 cm^-1^ in the substrate-bound form. The magnitude of this shift is 200 cm^-1^ less than in wildtype CAO1. As this decrease in energy reflects distortion toward trigonal bipyramidal geometry induced by substrate binding, the smaller magnitude of the shift indicates a more limited Fe^II^ geometric distortion. This difference in distortion was also manifested in the crystal structure of T151V Fe^II^–CAO1 in complex with piceatannol where a ∼6° compression of ∠His197N^ε^–Fe–H_2_O is observed *versus* a 21° compression of the corresponding angle in the wildtype enzyme. Inspection of the piceatannol binding mode in the T151V structure (Fig. 4*F*) revealed some small but notable differences compared with that of the wildtype enzyme that could be responsible for the different outcomes in iron center distortion. First, the β-ring of piceatannol is rotated ∼7° away from the Val151 side chain as expected based on the less favorable interaction between the piceatannol 3′-hydroxyl group and the non-native Val151 methyl group. As a possible consequence, the piceatannol molecule is shifted away from the iron center by ∼0.13 Å, which reduces steric interactions with the iron-bound solvent. This effect is enhanced by the shift of the iron away from the piceatannol binding site, thereby providing more space for the solvent to bind with less distortion away from planar geometry.

### Steady-state kinetics of wildtype and variants of CAO1

**Figure 5:**
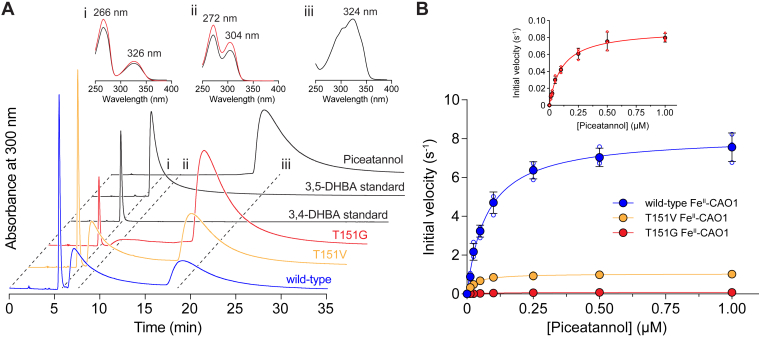
**Piceatannol cleavage specificity and steady-state kinetics of wildtype and occluding site–substituted Fe^II^–CAO1.***A*, HPLC chromatograms demonstrating the formation of 3,4-dihydroxybenzaldehyde (DHBA) and 3,5-DHBA from piceatannol for wildtype and variant Fe^II^–CAO1. The *insets* show absorbance spectra for the products formed by T151G Fe^II^–CAO1 together with reference spectra obtained from authentic standards. *B*, initial velocities as a function of piceatannol concentration are plotted for wildtype and occluding site–substituted Fe^II^–CAO1 together with least-squares fits to the Michaelis–Menten equation. The *inset* shows the data for T151G CAO1 on a magnified scale to illustrate the small but measurable activity for this variant. *Solid points* and *uncertainty bars* represent means and standard deviations from assays performed in triplicate. Individual data points are shown as *open circles*. In some cases, the error bars and individual data points are hidden behind the mean value symbols. Best-fit Michaelis–Menten parameters are given in Table 1. CAO1, *Neurospora crassa* carotenoid oxygenase 1.

To test the impact of occluding residue substitutions on CAO1 catalytic function, the enzymatic activities of wildtype, T151V, and T151G Fe^II^–CAO1 were characterized using piceatannol as a substrate (9, 12). Analyses of the reaction products by HPLC confirmed cleavage at the central α–β alkene bond in all cases (Fig. 5*A*). Steady-state kinetic assays were conducted over a range of piceatannol concentrations prepared in an air-saturated buffer (∼260 μM dissolved O_2_). As shown in Figure 5*B*, both substitutions reduced CAO1 catalytic function with the T151G substitution being especially detrimental. Least-squares fits of the Michaelis–Menten equation to the initial velocity data yielded the apparent steady-state kinetic parameters shown in Table 1. As compared with the wildtype enzyme, T151V and T151G CAO1 showed 2.8-fold and 134-fold reduced apparent catalytic efficiency toward piceatannol. The severe activity impairment observed for T151G CAO1 is attributable to its perturbed iron coordination geometry as shown by MCD spectroscopy and crystallography. By contrast, the CAO1 iron coordination number was unaffected by the T151V substitution, suggesting that the milder activity impairment for this variant could arise in part from the less favorable interaction between the 3′-hydroxyl moiety of piceatannol and the Val151 methyl group. To test this hypothesis, wildtype and T151V steady-state kinetics toward isoeugenol, which contains a 3′-methoxy group that could more favorably interact with the Val151 methyl group, were measured (Fig. S8). T151V CAO1 had ∼3.3-fold higher catalytic efficiency toward isoeugenol compared with wildtype CAO1 (Table 1) demonstrating that the T151V Fe^II^–CAO1 activity impairment is substrate dependent. Notably, isoeugenol was a far poorer substrate for both enzymes compared with piceatannol. These data demonstrate the importance of the occluding Thr residue for CAO1 catalytic function, both in regulating iron coordination geometry and in favorably interacting with the organic substrate. The particularly detrimental impact of the T151G substitution, which allows 6C iron ligation, suggests that the 5C resting state of the wildtype enzyme is pivotal for efficient catalysis.

**Table 1:** Wildtype and variant CAO1 apparent steady-state kinetic parameters

Parameter	Wildtype CAO1	T151V CAO1	T151G CAO1
*k*_cat_^app^ (s^-1^)	8.154 ± 0.215 (0.068 ± 0.004)	1.045 ± 0.024 (0.247 ± 0.012)	0.089 ± 0.003
*K*_*m*_^app^ (μM)	0.075 ± 0.007 (86.26 ± 13.49)	0.027 ± 0.003 (93.92 ± 12.27)	0.110 ± 0.013
*k*_cat_^app^/*K*_*m*_^app^ (s^-1^ μM^-1^)	108.72 ± 10.54 (7.88 × 10^-4^ ± 1.32 × 10^-4^)	38.70 ± 4.42 (2.63 × 10^-3^ ± 3.67 × 10^-4^)	0.81 ± 0.10

Values are the means ± standard errors. Values were obtained using piceatannol as the substrate except for the values in parentheses, which were obtained with isoeugenol as the substrate. Reactions were performed in air-saturated buffer.

### Correlating the carotenoid-cleaving dioxygenase *Nd*CCD to the stilbenoid cleaving CAO1

**Figure 6:**
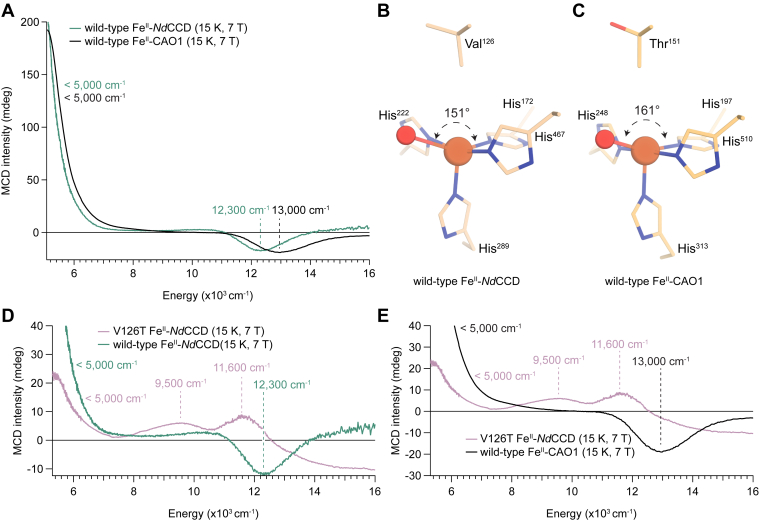
**Wildtype *Nd*CCD Fe^II^ site structure and impact of a V126T occluding site substitution.***A*, MCD spectrum of resting, wildtype Fe^II^–*Nd*CCD (*green*), and resting wildtype Fe^II^–CAO1 (*black*). *B*, crystal structure of resting Fe^II^–CAO1. *C*, crystal structure of resting Fe^II^–*Nd*CCD. *D*, MCD spectrum of resting wildtype Fe^II^–*Nd*CCD (*green*) and its resting V126T Fe^II^–*Nd*CCD (*purple*). *E*, MCD spectrum of resting V126T Fe^II^–*Nd*CCD variant (*purple*) and resting wildtype Fe^II^–CAO1 (*black*). The data in *A*, *D*, and *E* are representative of two independent experiments. CAO1, *Neurospora crassa* carotenoid oxygenase 1; MCD, magnetic CD; *NdCCD*, *Nitrosotalea devanaterra* carotenoid cleavage dioxygenase.

To determine whether the 5C square pyramidal structure of the resting Fe^II^ site is a general feature of both stilbenoid- and carotenoid-cleaving CCDs, NIR LT MCD spectroscopy was measured on an archaeal apocarotenoid-cleaving CCD, *Nd*CCD (8). The wildtype Fe^II^–*Nd*CCD NIR LT MCD spectrum shows two LF transitions, one at <5000 cm^-1^ and a second, weak negative transition at 12,300 cm^-1^ (Fig. 6*A*, *green*), resembling that of wildtype Fe^II^–CAO1 (*black*). In *Nd*CCD, the negative high-energy feature at 12,300 cm^-1^ is shifted down from 13,000 cm^-1^ in CAO1, suggesting that its iron center has a more trigonal bipyramidal–like structure. This geometric difference is confirmed in the crystallographic structure of Fe^II^–*Nd*CCD (8), which shows a reduction of ∠His172N^ε^–Fe–H_2_O to 151° from 161° in the corresponding angle of Fe^II^–CAO1 (Fig. 6, *B* and *C*). These data demonstrate a conserved 5C, distorted square pyramidal structure among diverse members of the CCD superfamily.

The contribution of the occluding site residue to the Fe^II^ site structure was further investigated by substituting Thr, as found in CAO1, for Val in *Nd*CCD. The NIR LT MCD spectrum of V126T *Nd*CCD shows three LF transitions at <5000, ∼9500, and ∼11,600 cm^-1^ (Fig. 6*D*, *purple*), where the negative shift at >12,000 cm^-1^ is field-induced baseline effect. The low-energy <5000 cm^-1^ transition reflects the presence of a 5C component, and the presence of at least two transitions at 9500 and 11,600 cm^-1^ indicates that there is also a 6C Fe^II^ component present. To characterize the additional species, the spectrum of V126T was fitted with four bands (Fig. S9*A*). The best fit to these data gives four features at 4900, 8330, 9560, and 11,500 cm^-1^. Fits to these data that included only three transitions necessitated an unrealistic full-width at half-maximum value for the transition at ∼9500 cm^-1^ (Fig. S9*B*). The energies of these four transitions are consistent with the presence of one 6C and one 5C component, a marked difference from wildtype Fe^II^–*Nd*CCD, which exhibits a single 5C site (Fig. 6*D*, *green*). Furthermore, this variant shows a different Fe^II^ site as compared with wildtype CAO1 (Fig. 6*E*, *black*) despite both iron centers sharing common first- and second-sphere coordination residues.

The catalytic impact of the V126T substitution was assessed through steady-state kinetic analysis. The catalytic activity of the V126T variant toward the substrate all-*trans*-β-apo-8′-carotenal was markedly impaired relative to the wildtype enzyme, although the product specificity was maintained in the mutant enzyme (Fig. S10). Determination of the Michaelis–Menten parameters showed a 93% reduction in catalytic efficiency (*k*_cat_/*K*_*m*_) for V126T Fe^II^–*Nd*CCD relative to the wildtype enzyme (Table S4). The structural and catalytic variation of the V126T Fe^II^–*Nd*CCD mutant provides further evidence that the occluding site plays an important role in CCD Fe^II^ coordination geometry and catalytic activity (32).

## Discussion

The spectroscopic, crystallographic, and kinetic analyses presented above: (1) describe the effect of substrate binding on the activation of the CAO1 Fe^II^ site for reactivity with NO and potentially also O_2_; (2) provide insights into the mechanism of O_2_ binding to the substrate-bound site; (3) identify the necessity of the semiconserved, occluding second-sphere residue for proper reactivity; and (4) give insight into the evolutionary selection of the occluding second-sphere residue in individual CCD enzymes.

MCD spectroscopy and crystallography defined the structure of the resting Fe^II^ site in the stilbenoid-cleaving CAO1 as 5C, square pyramidal, which results from the occlusion of the sixth coordination position by a second-sphere Thr residue. Our MCD spectroscopic structural definition supports the previous crystallographic characterization of diverse members of the CCD superfamily (2, 8, 9, 10, 33, 34). The resting Fe^II^ site, despite being 5C, is stable in the presence of O_2_ (9), in contrast to previous proposals suggesting that CCDs can bind O_2_ in the absence of substrate (21, 22, 23) that implied unsynchronized reductive activation of the bound O_2_ (35). The stability of the resting Fe^II^ site to O_2_ is due in part to the 4-His coordination environment, which is less electron donating relative to the facial triad ligation. This study also defines the role of the substrate in activating the Fe^II^ site for reactivity. We find that the CAO1 Fe^II^ site undergoes a distortion away from square pyramidal geometry because of steric interaction of the substrate with an iron-coordinated water, resulting in its repositioning toward the open coordination position. To understand the potential effect of this distortion on O_2_ binding, we found that the substrate-bound Fe^II^ site has more than two orders of magnitude greater affinity for the O_2_ analog NO as compared with the resting site. Together, these data indicate a mechanism where the change of geometry that is induced by the steric interaction of the substrate and water activates the Fe^II^ site for reaction with NO and potentially O_2_.

Previous computational studies have described two possible modes of initial O_2_ binding: (1) O_2_ binds associatively to the iron without loss of the solvent ligand or (2) O_2_ replaces the solvent (17). Our study elucidates critical roles for both the second-sphere occluding residue and the substrate in determining Fe^II^ site coordination and reactivity. We find that while the resting Fe^II^ site in the less constrained T151G CAO1 variant is 6C, substrate binding induces loss of a water ligand in a fraction of sample, which appears to confer higher binding affinity for NO. In addition, in wildtype Fe^II^–CAO1, substrate binding induces a steric distortion to the bound solvent, which is correlated with a significant increase in NO affinity. These results suggest that the combined steric interaction of the substrate and the second-sphere occluding residue with the adjacent water ligand led to its replacement by the O_2_ analog NO. The Fe^II^ site sixth coordination position is completely occluded in the resting structure of wildtype Fe^II^–CAO1, and it remains occluded in the piceatannol-bound structure. Hence, for an associative mechanism to form a 6C site, the Thr side chain would need to adopt an alternative conformation to allow the necessary space for the solvent. However, the Thr side chain is locked into the observed rotamer by a combination of hydrogen bonding interactions and steric factors, making other potential rotamers inaccessible (2, 9). Other possible space-liberating conformational variability is unlikely since the portion of the protein chain containing the Thr151 residue is deeply buried and positionally restrained. Moreover, inspection of the numerous CCD crystal structures determined to date shows that the side chain methyl group is invariably directed toward the sixth coordination site, acting as an occluding “plug” (36). These considerations, together with the present experimental data, strongly favor a mechanism whereby the bound solvent is exchanged for O_2_ following ES complexation. Notably, neither the MCD data nor the crystallographic results show evidence for substrate-induced formation of a putative 4-coordinate intermediate.

The aforementioned results raised the question of whether the 5C geometry of CCD enzymes is important for efficient catalytic function. In this study, we used a series of occluding site mutants in both stilbenoid- and carotenoid-cleaving enzymes to assess the functional role of the specific residue at the occluding site. In the stilbenoid-cleaving CAO1, the volume of the side chain was maximally reduced by making a Thr to Gly substitution. Both spectroscopic and crystallographic characterization of this T151G variant revealed that the resting Fe^II^ site adopts a 6C, distorted octahedral geometry, where an additional solvent molecule occupies the position left vacant in the wildtype enzyme. This mutation demonstrates the key role that the occluding residue plays in the wildtype structure to sterically impose a 5C geometry. Steady-state kinetics show that the T151G substitution results in a >100-fold decrease in catalytic efficiency compared with the wildtype enzyme. This activity reduction demonstrates the necessity of the 5C geometry for reactivity, with the low residual activity of the T151G variant potentially attributed to the 5C component that forms upon substrate binding. The minimal perturbation in binding orientation of the organic substrate caused by the T151G substitution indicates that the catalytic impairment is directly caused by the iron center structural change, which could impact productive binding and activation of O_2_ or its reaction with the substrate because of the presence of the extra iron-coordinated solvent.

Additional occluding-site substitutions were made to both the stilbenoid-cleaving CAO1 and the carotenoid-cleaving *Nd*CCD. We found that T151V Fe^II^–CAO1 catalytic activity toward piceatannol is impaired, likely because of an alteration in the positioning of the substrate within the active site pocket, a decreased distortion of the substrate-bound Fe^II^ center, or a combination of the two contributions. Similarly, V126T Fe^II^–*Nd*CCD exhibited a mixture of 5C/6C Fe^II^ sites and greatly reduced catalytic activity. In addition to the perturbing effect of the V126T substitution on the iron center, the Thr hydroxyl moiety likely interacts unfavorably with the hydrophobic apocarotenoid substrate of *Nd*CCD (8). Although an occluding methyl group appears to be a conserved feature of the CCD Fe^II^ center, the results presented here indicate that the specific residue used within a particular subfamily is under strong selective pressure for maintenance of catalytic function. Sequence analysis indicates that among CCD subfamilies the occluding residue is usually strongly conserved (Fig. S1). For example, within the stilbenoid-cleaving clade of CCDs, a Thr residue is invariably found at the occluding site. Our structural data on Fe^II^–CAO1–substrate complexes indicate the Thr side chain serves a dual purpose in occluding the iron coordination site as well as favorably interacting with catechol-containing substrates like piceatannol. Interestingly, T151V Fe^II^–CAO1 exhibited greater activity toward isoeugenol compared with the wildtype enzyme, possibly because of a more favorable interaction of the 3′-methoxy group of isoeugenol with the Val side chain compared with the hydroxyl group of Thr.

One potential exception to the otherwise widespread presence of an iron-occluding Thr/Val/Ile residue in CCDs is the 9-*cis*-epoxycarotenoid dioxygenase subfamily, exemplified by maize viviparous-14, which possesses an Ala residue at the occluding site (Fig. S1). The viviparous-14 crystal structure was modeled with a 6C iron center containing bound O_2_ and hydroxide in the two exchangeable coordination positions (22). However, the resolution and quality of the diffraction data supporting this structure were limited (∼3.2-Å resolution, Wilson *B*-factor >100 Å^2^) making the presence of these ligands in the iron coordination sphere uncertain. Indeed, a Thr to Ala substitution in *Synechocystis* apocarotenoid oxygenase was insufficient to produce a 6C iron site (5). Further studies are needed to clarify the iron coordination number and geometry in the 9-*cis*-epoxycarotenoid dioxygenases.

In conclusion, we define the CCDs as a new subclass of the non–cofactor-dependent, non-heme iron(II) oxygenases having a 4-His/H_2_O 5C Fe^II^ site with an open coordination position maintained by an occluding second sphere residue (Table S1). The data presented here in conjunction with prior structural and spectroscopic studies support a catalytic model in which substrate binding geometrically perturbs the Fe^II^ site promoting exchange of the bound water for O_2_, which can then react with the conjugated alkene substrate. The data help resolve a long-standing controversy related to the capacity of CCDs to bind O_2_ in the absence of their organic substrates and provide key information needed to advance mechanistic understanding of CCD catalysis.

## Experimental procedures

### Expression and purification of Fe^II^–CAO1, Fe^II^–*Nd*CCD, and active site mutants

Wildtype CAO1 and *Nd*CCD were expressed as previously described (8, 9). Plasmids encoding T151V and T151G CAO1 and V126T *Nd*CCD were generated by site-directed mutagenesis and verified by Sanger sequencing. The wildtype and occluding site–substituted proteins were purified as previously described (8, 9), with the exception that 20 mM Hepes–NaOH, pH 8 was used as the buffer for the initial anion exchange chromatography step in CAO1 purifications. The purified proteins were concentrated to 50 to 150 mg/ml, flash frozen in liquid nitrogen, and stored at −80 °C until needed. Iron loading of the purified proteins was performed as described previously (37).

### Crystallization of wildtype and mutant CAO1 and formation of piceatannol complexes

Crystallization of wildtype Fe^II^–CAO1, T151V Fe^II^–CAO1, and T151G Fe^II^–CAO1 was performed by the hanging-drop vapor-diffusion method as described previously reported for wildtype CAO1 (9). Two milliliters of purified protein at a concentration of 20 mg/ml was added to an equal volume of crystallization solution containing 42 to 43% (w/v) sodium polyacrylate 2100 (Sigma–Aldrich) and 0.05 to 0.1 M Hepes–NaOH, pH 7.0, in a 1:1 ratio. The resulting drops were placed in a sealed chamber over 0.5 ml of the crystallization solution. The crystallization plates were then incubated at 4 or 21 °C. Rod-shaped crystals appeared within 2 to 4 days and grew to full size (100 × 100 × 600 μm) within 1 to 2 weeks. Mature wildtype, T151V, and T151G CAO1 crystals were transferred into crystallization solution containing 10 mM piceatannol (TCI), which was prepared by adding 1 μl of a 1 M piceatannol stock in dimethyl sulfoxide (DMSO) to 99 μl of crystallization solution. The crystals were allowed to soak for various time intervals before harvesting (4 min at room temperature for wildtype CAO1, 2 min at room temperature for T151V CAO1, and 5 h at 16 °C for T151G CAO1). Crystals were harvested and flash frozen in liquid nitrogen using MiTeGen dual-thickness loops. The crystals were stored in liquid nitrogen and shipped in liquid nitrogen vapor for X-ray data collection.

### Diffraction data collection, structure determination, and refinement

X-ray diffraction data were collected at the NE-CAT 24-ID-C and 24-ID-E beamlines of the Advanced Photon Source. Crystals of T151V and T151G Fe^II^–CAO1 mutants were isomorphous to wildtype Fe^II^–CAO1 crystals, belonging to space group *P*3_2_21 with four monomers in the asymmetric unit. Diffraction data reduction was performed with XDS (38), and the structures were determined by direct refinement in REFMAC (39) using the coordinates of wildtype Fe^II^–CAO1 (Protein Data Bank [PDB] accession code: 5U8X) as a starting model. The models were improved by iterative cycles of manual model building and adjustments in COOT (40) followed by reciprocal space refinement in REFMAC (39) including occupancy refinement of the active-site metal, metal-bound waters, and piceatannol ligands. The model geometry and fit to the electron density maps were evaluated using the MOLPROBITY (41) and wwPDB (42) validation servers. To allow accurate comparisons to the newly reported structures from this work, the structure of wildtype Fe-CAO1 (PDB accession code: 5U8X) was rerefined in REFMAC using the standard CCP4 piceatannol ligand dictionary file and with the active-site iron and iron-bound water subjected to occupancy refinement. The updated coordinates were redeposited in the PDB under the same accession code. Images of the structures were prepared using PyMOL (Schrödinger, Inc).

### HPLC analysis of CAO1 wildtype and mutant enzymatic activity

Reactions were performed in 10 mM Tris–HCl (pH 7.0) in a total volume of 100 μl placed inside 1.5 ml amber microcentrifuge tubes (Thermo Fisher Scientific). A stock solution of piceatannol was prepared in DMSO (Fisher Scientific). Purified protein samples of wildtype, T151V, and T151G CAO1 were incubated with 30 μM piceatannol at 28 °C in a shaker-incubator running at 300 RPM. After 30 min, 300 μl of 100% ethyl acetate (Fisher Scientific) were added to each reaction tube followed by vigorous shaking for 2 min to extract the substrates and products. The tubes were centrifuged at 21,130*g* for 4 min in a tabletop centrifuge. The organic layer was transferred to a borosilicate tube and dried in a SpeedVac concentrator (Thermo Scientific). The dried substrate and cleavage products were redissolved in 200 μl of 60:40 hexanes/ethyl acetate and transferred to flat-bottomed glass inserts placed inside amber wide crimp top vials and sealed with silver crimp PTFE/rubber caps. The samples were analyzed by normal-phase HPLC on an Agilent 1100 Series instrument equipped with a diode array detector and an Agilent Zorbax SIL, 4.6 mm ID × 250 mm (5 μm) column that was prewashed and equilibrated with a mobile phase of 60:40 hexanes/ethyl acetate. Samples were eluted with the same mobile phase at a flow rate of 1.4 ml/min. Chromatogram signals measured at 300 nm were plotted using GraphPad software along with the absorption spectra for the substrate, products, and authentic standards (Sigma–Aldrich).

### CAO1 steady-state kinetic measurements

Steady-state activity measurements were performed using piceatannol as a substrate in an air-saturated buffer containing 20 mM Hepes–NaOH and 1 mM Tris(2-carboxyethyl)phosphine, pH 7.2. Reactions were monitored spectrophotometrically by recording the time-dependent decrease in sample absorbance at 324 nm arising from the enzymatic cleavage of the central alkene bond in piceatannol. Reactions were performed in a long pathlength (5 cm) cuvette (Firefly Sci, 29UV50) to enable reliable measurement of submicromolar substrate concentrations. A 100 mM stock of piceatannol in DMSO was diluted in the reaction buffer to achieve concentrations in the final reaction volume ranging from 0.0125 to 1 μM. Final concentrations of iron-loaded wildtype, T151V, and T151G CAO1 were 0.1, 1, and 14.4 nM, respectively. After confirming a stable absorbance reading for the reaction buffer containing substrate at a given concentration, the reaction was initiated by the addition of enzyme followed by gentle mixing. After a ∼10 s period to allow the absorbance to stabilize following mixing, the decrease in absorbance at 324 nm was recorded every 6 s for 30 to 60 s, over which time the decline in absorbance was linear. A standard curve was used to determine the extinction coefficient for piceatannol in the reaction buffer at 324 nm (ε_324_ = 31,600 M^-1^ cm^-1^) allowing conversion of absorbances to concentrations. The initial velocities (*V*_*o*_) were then plotted as a function of substrate concentration (*S*), and the steady-state parameters were extracted by fitting to the Michaelis–Menten equation:

$$V_{o}=\frac{V_{\max}\times S}{K_{m}+S}$$

where *V*_max_ is the maximal velocity and *K*_*m*_ is the substrate concentration at half-maximal velocity. Velocities were divided by the concentration of iron-loaded enzyme used in the assay to obtain turnover numbers.

Assays using isoeugenol as a substrate were performed similarly except that substrate concentrations of 6.25 to 500 μM and an enzyme concentration of 120 nM for both wildtype and T151V Fe^II^–CAO1 were used. The increase in absorbance at 350 nm, reflecting formation of vanillin product, was monitored over time. The vanillin extinction coefficient, ε_350_ = 9260 M^-1^ cm^-1^ in reaction buffer, was used to convert absorbance values into concentrations.

### *Nd*CCD steady-state kinetic measurements

The assays were performed under dim red light to prevent unwanted photoisomerization reactions. The reaction buffer consisted of 250 μl of 20 mM Bis–Tris–HCl (pH 7.0), 0.05% Triton X-100, 10 μg of wildtype *Nd*CCD, or 40 μg of V126T *Nd*CCD and a range of all-*trans*-β-apo-8′-carotenal concentrations. The reactions were carried out for 15 min at room temperature, which was confirmed to be within the linear range of product formation and then quenched with 100 μl of methanol. After quenching, 300 μl of brine and 500 μl of hexanes were added and mixed vigorously for 2 min. The solution was then centrifuged at 16,000*g* for 3 min. Immediately after centrifugation, 200 μl of the top organic layer were removed and placed into an amber HPLC vial containing a borosilicate insert and capped. The samples were analyzed on a 1260 Infinity II series HPLC (Agilent) equipped with a diode array detector and a Zorbax XR-SIL 5 μm column (Agilent). The substrate and product were eluted with a 90:10 hexane/ethyl acetate mobile phase at a flow rate of 1.4 ml/min. A calibration curve was generated using known quantities of all-*trans*-β-apo-14′-carotenal to allow conversion of product peak areas to masses. The initial velocities were then plotted as a function of substrate concentration, and the steady-state parameters were extracted by fitting to the Michaelis–Menten equation as described previously.

### Preparation of spectroscopic samples

All samples for spectroscopic measurement were prepared in an anaerobic environment. The enzyme samples were deoxygenated by flowing ultra-high purity nitrogen gas through the headspace of a stirred solution (4 °C) in a sealed vial. The samples were then transferred into an anaerobic, inert N_2_-atmosphere wet box. Buffer solutions (CAO1: 20 mM Hepes–NaOH, 150 mM NaCl, pH 7.2 or pD 6.76, *Nd*CCD: 20 mM Hepes–NaOD, pH 7.2 or pD 6.76) and 200 mM piceatannol in DMSO (CAO1 substrate) were made anaerobic by flowing ultra-high purity nitrogen gas through the headspace of the solutions in sealed vials before being transferred in the inert N_2_-atmosphere wet box. In an anaerobic chamber, the anaerobic enzyme was reduced with a 220 μM sodium dithionite solution made in anaerobic buffer. The protein was incubated with 15 M equivalents, with stirring, for 1.5 h to ensure complete conversion to the reduced Fe^II^ form. This was necessary to prevent contaminant signals in the near-IR and UV–visible regions. For measurement of samples in the near-IR region and to remove residual sodium dithionite, the fully reduced enzyme samples were then exchanged into deuterated buffer (D_2_O; Sigma–Aldrich 99.9%) and concentrated (CAO1: 20 mM Hepes–NaOD, 150 mM NaCl, pD = 6.76, *Nd*CCD: 20 mM Hepes–NaOD, pD = 6.76) using 30 kDa cutoff Amicon Ultra-0.5 ml centrifugal filters (Millipore). Enzyme concentrations ranged from 3 to 6 mM in deuterated buffer solutions. For samples at pH 8.5, the enzyme was exchanged into deuterated buffers at 20 mM Hepes–NaOD, 150 mM NaCl, pD 8.06 and samples at pH 9.5, the enzyme were exchanged into deuterated buffers at 20 mM Hepes–NaOD, 150 mM NaCl, pD 9.1.

For MCD measurements, Fe^II^–CAO1 and Fe^II^–*Nd*CCD samples were 3.4 mM Fe^II^ enzyme. ES complex samples were 3.4 mM Fe^II^–CAO1 and 3.4 mM piceatannol. High-quality optical glasses were obtained by adding solid *d*_*8*_-sucrose until saturated. Samples were transferred into an optical MCD cell, comprised of two Infrasil quartz disks separated by a 2.5 mm Viton O-ring spacer, clamped together into a copper metal holder. The cells were then transferred from the glovebox and then frozen immediately in liquid nitrogen for measurement.

### MCD spectroscopic measurements of CAO1 and *Nd*CCD

MCD spectra were measured using a JASCO J-1700 polarimeter (300–2000 nm). The spectropolarimeter is coupled with an 8 ft sample compartment housing a liquid helium–cooled Oxford Instruments SM-4000 7 T superconducting magnet between the polarimeter source and detector. MCD sample temperature was measured using a Lake Shore Cryotronics Cernox sensor (calibrated for 1.4–325 K) inserted directly into the sample cell. MCD spectra were acquired (average of 5–10 scans) at temperatures of 2, 5, 15, and 25 K for fields of ± 7, ± 5, ± 3, ± 1, and 0 T. Baseline-corrected spectra were obtained as the difference of positive- and negative-field scans divided by two from the zero-field scans to eliminate zero-field contributions.

### UV–visible–NIR antibodies and CD measurements of Fe^II^–CAO1

For the substrate titration UV–visible absorption and CD measurements, CAO1 was deoxygenated, reduced, and exchanged into deuterated buffer in the same steps outlined for MCD cell preparations. Enzyme solutions were made in airtight cuvettes for measurement. To determine the *K*_*d*_ of piceatannol binding to wildtype Fe^II^–CAO1, 20 μM of Fe^II^–CAO1 was titrated with 5 μM piceatannol aliquots while monitoring the piceatannol charge–transfer transition in the UV region. Additions of substrate were performed in an anaerobic glovebox. Substrate equivalents were added to the enzyme solutions in the airtight cuvette and allowed to bind for 5 min at 4 °C. The cuvette was then transferred from the glovebox to perform measurement. Simultaneous UV–visible (250–800 nm) absorbance and CD spectra were collected on a JASCO J-1700 spectropolarimeter in its standard configuration. All spectra were collected (five scans) at 4 °C and baseline corrected using the enzyme in buffer solution.

For the NO titration measurements, CAO1 was deoxygenated, reduced, and exchanged into deuterated buffer with 20% w/v sucrose to increase protein stability upon NO addition. To determine the *K*_*d*_ of NO binding to the Fe^II^–CAO1 resting and substrate-bound complexes, 200 or 400 μM wildtype Fe^II^–CAO1 (with excess piceatannol for ES complexes) was titrated with increasing equivalents of NO (0.125x, 0.25x, 0.5x, 1x, 3x, 5x, 10x, and 20x) from a stock solution of 80 mM MAHMA NONOate (Sigma–Aldrich) in pH = 10 NaOH. Additions of NO were performed in an anaerobic glovebox. NO equivalents were added to the enzyme solutions in the airtight cuvette and allowed to react for 5 min at 4 °C. The cuvette was then transferred from the glovebox to perform measurements. Simultaneous UV–visible (250–800 nm) absorbance and CD spectra were collected on a JASCO J-1700 spectropolarimeter in its standard configuration. All spectra were collected (five scans) at 4 °C and baseline corrected using the enzyme in buffer solution. Each titration was performed three times.

Absorption (and CD where applicable) titration data were fit to extract the *K*_*d*_ for both piceatannol binding to wildtype Fe^II^–CAO1 and NO binding to wildtype and T151G Fe^II^–CAO1. The titration data were fit to the following equation as previously described (43).

$$A=\frac{\varepsilon }{2}\left(\left(K_{d}+c+M\right)-\sqrt{{\left(K_{d}+c+M\right)}^{2}-4\times c\times M}\right)$$

A is the absorption intensity at the selected wavelength with the corresponding molar absorption coefficient ε, c is the concentration of the protein used in the titration, M is the concentration of either substrate or NO at each titration equivalent. In some cases, NO titration data were fit to the following two-site binding equation.

$$A=\frac{\varepsilon }{2}\left[\left(\left(K_{d1}+c_{1}+M\right)-\sqrt{{\left(K_{d1}+c_{1}+M\right)}^{2}-4\times c_{1}\times M}\right)+\left(\left(K_{d2}+c_{2}+M\right)-\sqrt{{\left(K_{d2}+c_{2}+M\right)}^{2}-4\times c_{2}\times M}\right)\right]$$

The numbers in the subscripts refer to the two different components. The piceatannol binding data were fit at 385 nm, the enzyme-only NO binding data were fit at 425 nm, and the ES–NO binding data were fit at 550 nm or 670 nm.

## Data availability

Data are contained within the article. Crystallographic data are deposited in the PDB under accession codes 5U8X, 8FU5, 7T8P, 8SRL, 7T8Q, and 8FU2.

## Supporting information

This article contains supporting information (9, 44, 45, 46, 47, 48, 49).

## Conflict of interest

The authors declare that they have no conflicts of interest with the contents of this article.
