# Supplementary material for: Spectroscopy and crystallography define carotenoid oxygenases as a new subclass of mononuclear non-heme FeII enzymes
Source: J Biol Chem. 2025 Mar 25;301(5):108444. doi: 10.1016/j.jbc.2025.108444 (PMC12051055; doi:10.1016/j.jbc.2025.108444)
Supplement: Supporting information [file mmc1.pdf]

Supporting Information

Spectroscopy and crystallography define carotenoid oxygenases as a new subclass of mononuclear non-heme Fe^{II} enzymes

Dory E. DeWeese[1], Michael P. Everett[2], Jeffrey T. Babicz Jr.[1,3], Anahita Daruwalla[2], Edward I. Solomon[1,3,*], and Philip D. Kiser[2,4,*]

[1] Department of Chemistry, Stanford University, Stanford, California 94305, USA

[2] Department of Physiology & Biophysics, University of California, Irvine School of Medicine, Irvine, CA, 92697, USA

[3] SLAC National Accelerator Laboratory, Stanford University, Menlo Park, California 94025, USA

[4] Research Service, VA Long Beach Healthcare System, Long Beach, CA, 90822, USA

* To whom correspondence should be addressed: solomone@stanford.edu or pkiser@uci.edu

Contents

Supplementary Figures 1-10

Supplementary Tables 1-4

Supplementary References

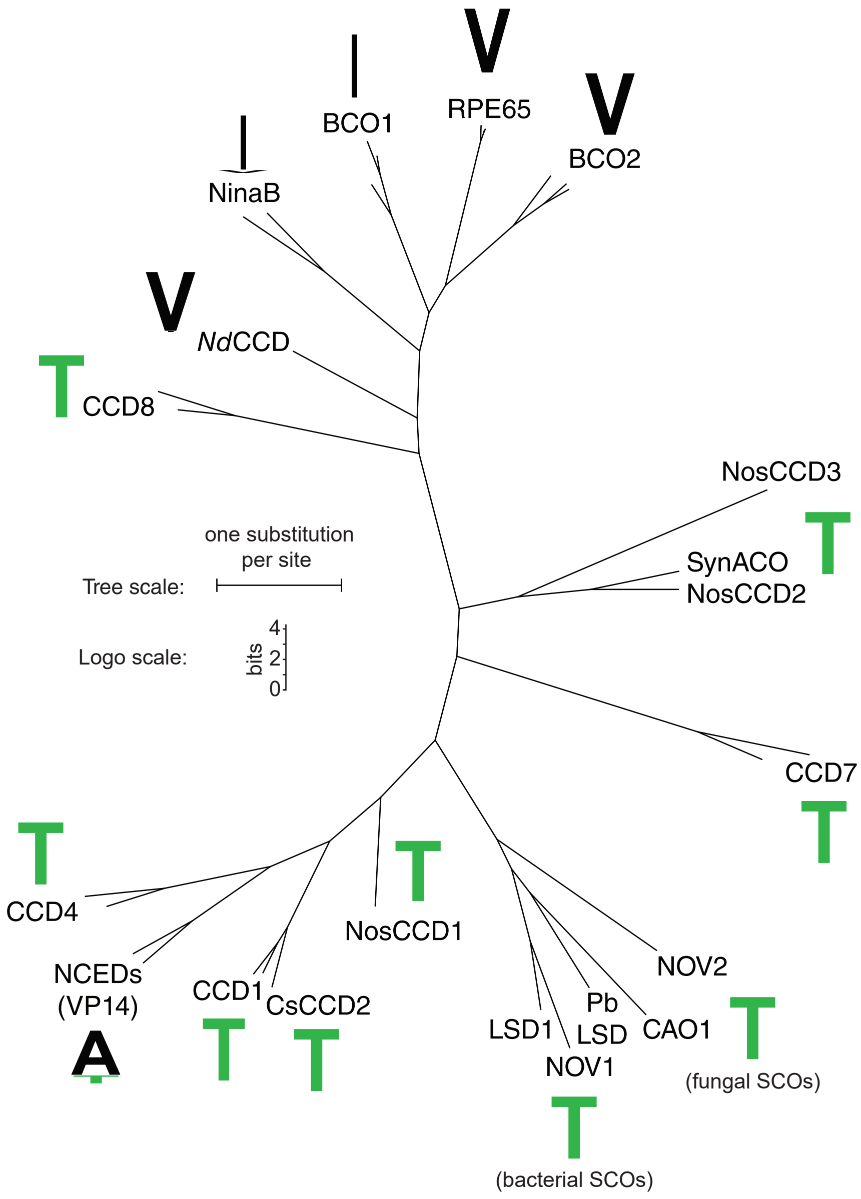

Figure S1: *Phylogeny of the CCD superfamily and occluding residue conservation.* The phylogenetic tree was constructed using PhyML [1] based on amino acid sequences aligned with MUSCLE. [2] The letters beside different subfamilies of CCDs are sequence logos [3] for the occluding site in each group. The logos were generated from >100 aligned, putatively orthologous, sequences obtained through NCBI Blast [4] searches using WebLogo. [5] The polar Thr residue is shown in green while non-polar side chains are shown in black. The sequence logos demonstrate the high degree of occluding site conservation within CCD subfamilies.

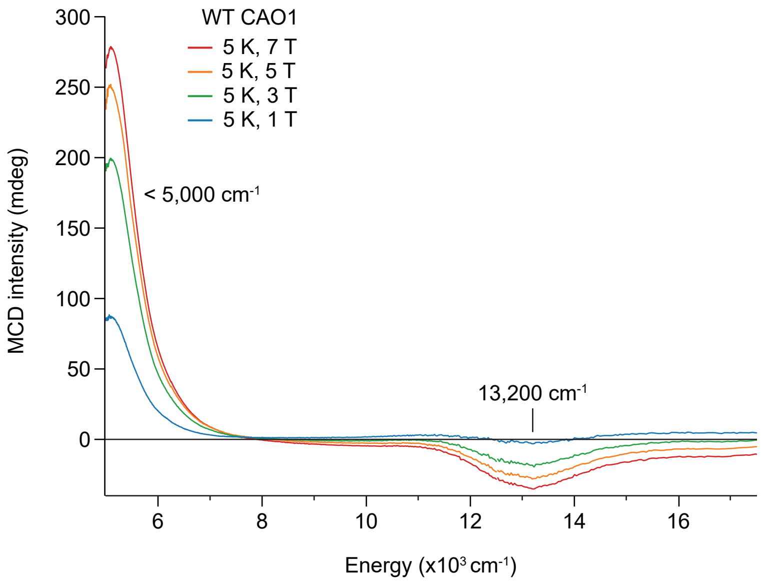

Figure S2. *Field-dependent MCD spectra of resting wild-type Fe^{II}-CAO1 measured at 5 K from 1 T to 7 T.* Both features at <5,000 cm^{-1} and ~13,000 cm^{-1} show field-dependent behavior typical of a paramagnetic ground state.

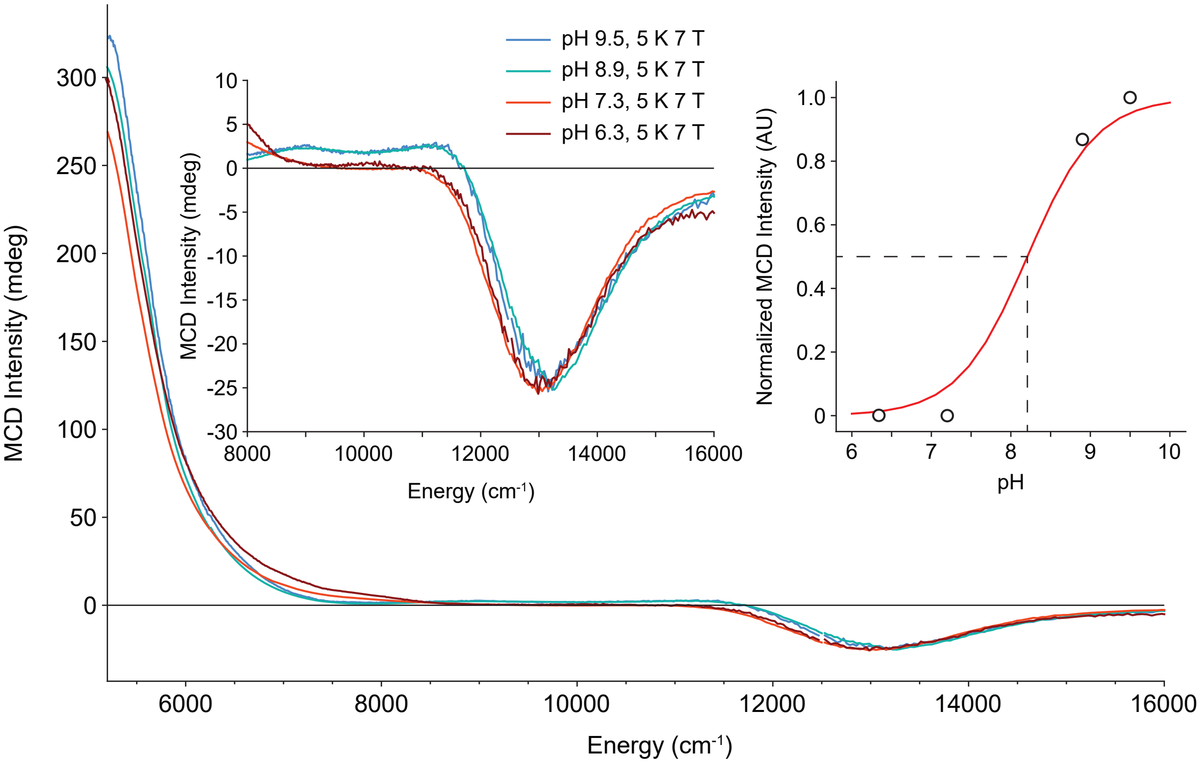

Figure S3. *Effect of pH on the resting Fe^{II}-CAO1 site measured by MCD* **A**) pH-dependent MCD spectra of wild-type Fe^{II}-CAO1 measured at pH 6.3 (red), 7.2 (orange), 8.9 (teal) and 9.5 (blue) at 5 K and 7 T. The left inset focuses on the 8,000 to 16,000 cm^{-1} region to show weaker features in the spectra. The right inset shows normalized MCD intensity (nMCD$_I$) at 9,000 cm^{-1} vs. pH, which was fit to the Henderson-Hasselbach equation, $nMCD_I = \frac{1}{1+10^{(pKa-pH)}}$, to give an estimate for the pK$_a$ of the bound water, where the best fit indicated a pK$_a$ of 8.2.

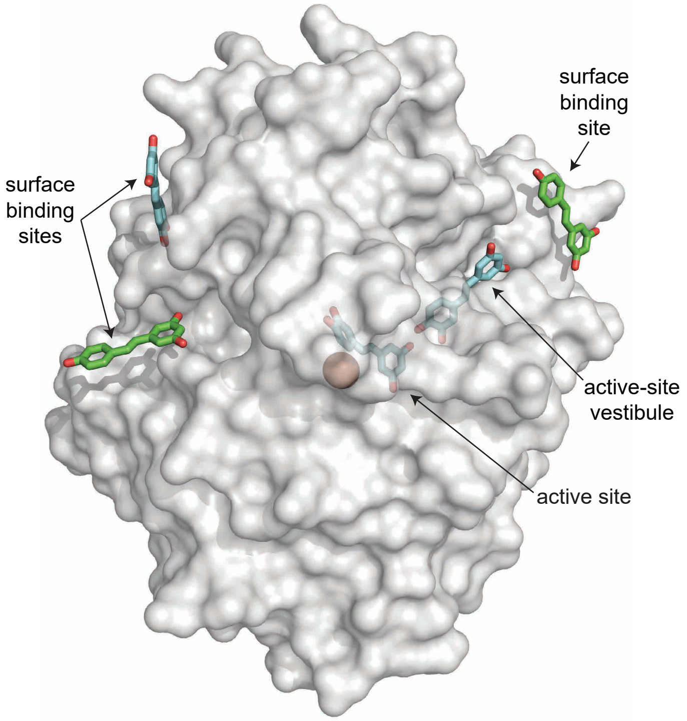

Figure S4. *Crystallographically observed stilbenoid-binding sites located on the surface, the active-site vestibule, and the active site of CAO1.* This composite figure was generated from PDB accession codes 5U90, 5U97, and 8FU2.

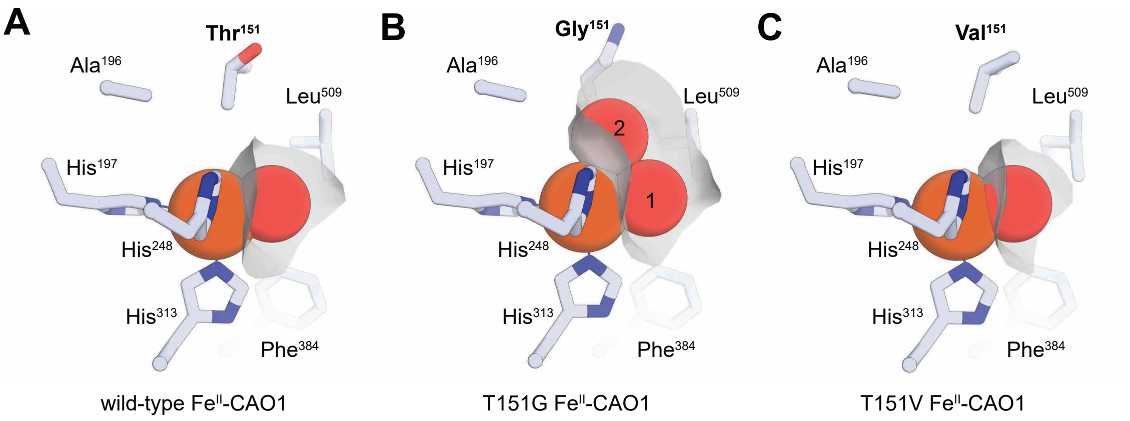

Figure S5. *Solvent-excluded surfaces near the iron center of wild-type and variant CAO1.* **A**) Wild-type Fe^{II}-CAO1 showing an occluded sixth site. **B**) T151G Fe^{II}-CAO1 exhibits an open, solvent-occupied sixth site. **C**) T151V Fe^{II}-CAO1 showing an occluded sixth site.

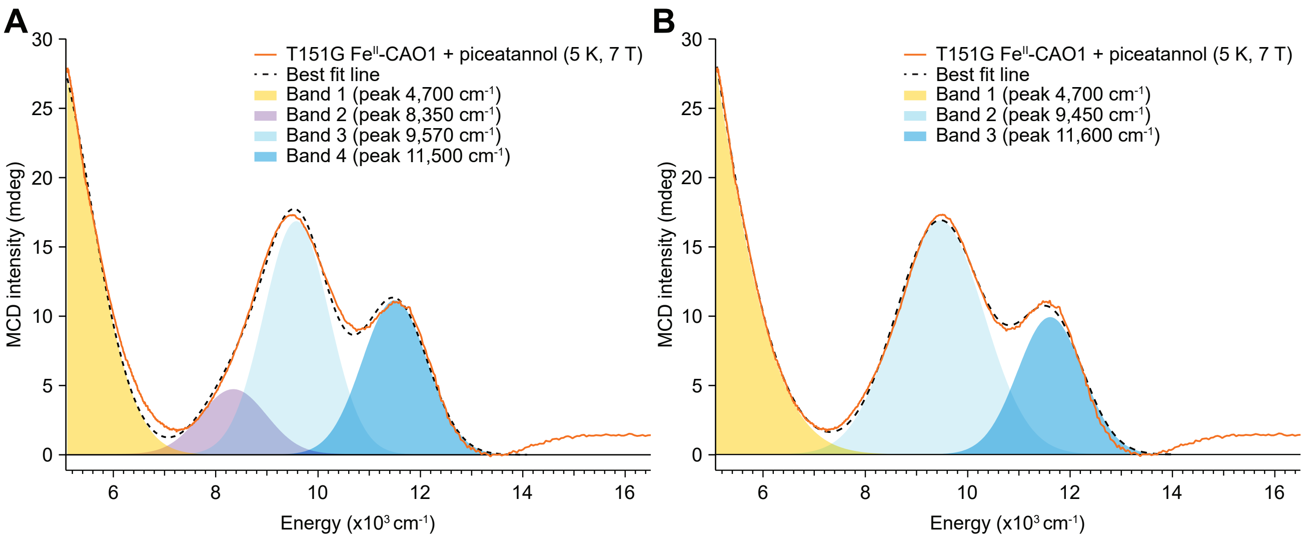

Figure S6. *Gaussian fitting of the T151G Fe^{II}-CAO1-piceatannol complex MCD data.* **A**) Best fit of the T151G substrate-bound low temperature, high field (5 K, 7 T) MCD data. The data are shown in orange, the best fit in dashed black line, and individual fits for the features are shown in yellow (band one at 4,700 cm^{-1}), purple (band two at 8,350 cm^{-1}), light blue (band three at 9,570 cm^{-1}) and dark blue (band four at 11,500 cm^{-1}). The four features were fit with Gaussian line shapes at a full-width, half-maximum (FWHM) value of 750 cm^{-1}, as set by the FWHM of the T151G CAO1 resting-state features. **B**) Fit of the T151G substrate-bound, low-temperature, high-field (5K, 7T) MCD data to three Gaussian features. The data are shown in orange, the best fit in dashed black line, and individual fits for the features are shown in yellow (band one at 4,700 cm^{-1}), light blue (band two at 9,450 cm^{-1}) and dark blue (band three at 11,600 cm^{-1}), where optimal fitting of the feature at 9,450 cm^{-1} necessitates an unrealistically large FWHM of 1,000 cm^{-1}.

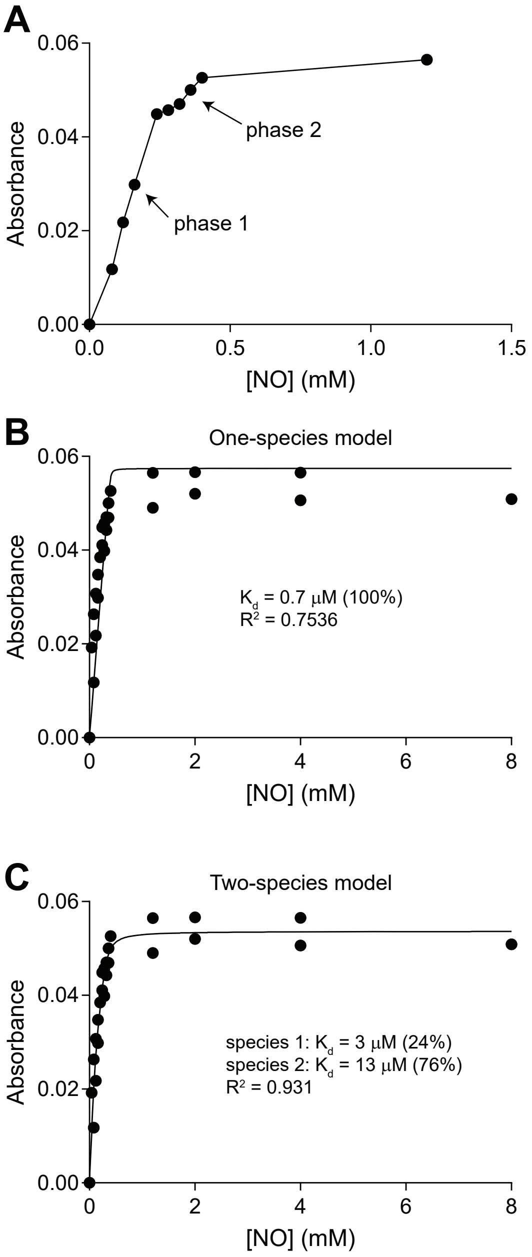

Figure S7. *NO titrations into the T151G Fe^{II}-CAO1 ES complex.* **A**) A single titration data set showing the biphasic appearance of the titration curve. **B**) The titration data were fit with a one-species quadratic binding equation, resulting in a visibly poor fit to the higher NO concentration data points and a relatively low R^2 value (0.7536). **C**) The same data were fit with a two-species quadratic binding equation, which provided a more satisfactory fit as evidenced by the improved R^2 value of 0.931.

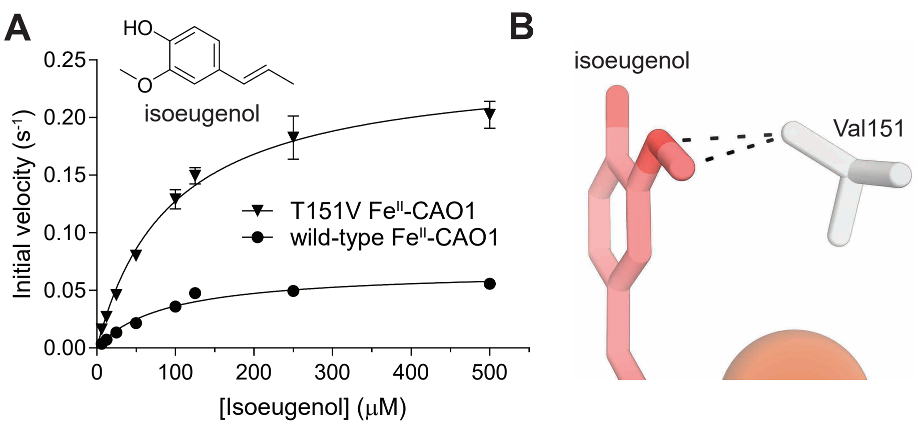

Figure S8. *CAO1 steady-state catalytic activity towards isoeugenol.* **A**) Initial velocities as a function of isoeugenol concentration are plotted for wild-type and T151V Fe^{II}-CAO1 together with least-squares fits to the Michaelis-Menton equation. The inset shows the chemical structure of isoeugenol. Best-fit Michaelis-Menton parameters are given in **Table S4**. Data points and uncertainty bars represent represents means and standard deviations. **B**) A hypothetical structural model of isoeugenol interacting with the Val151 residue of T151V Fe^{II}-CAO1.

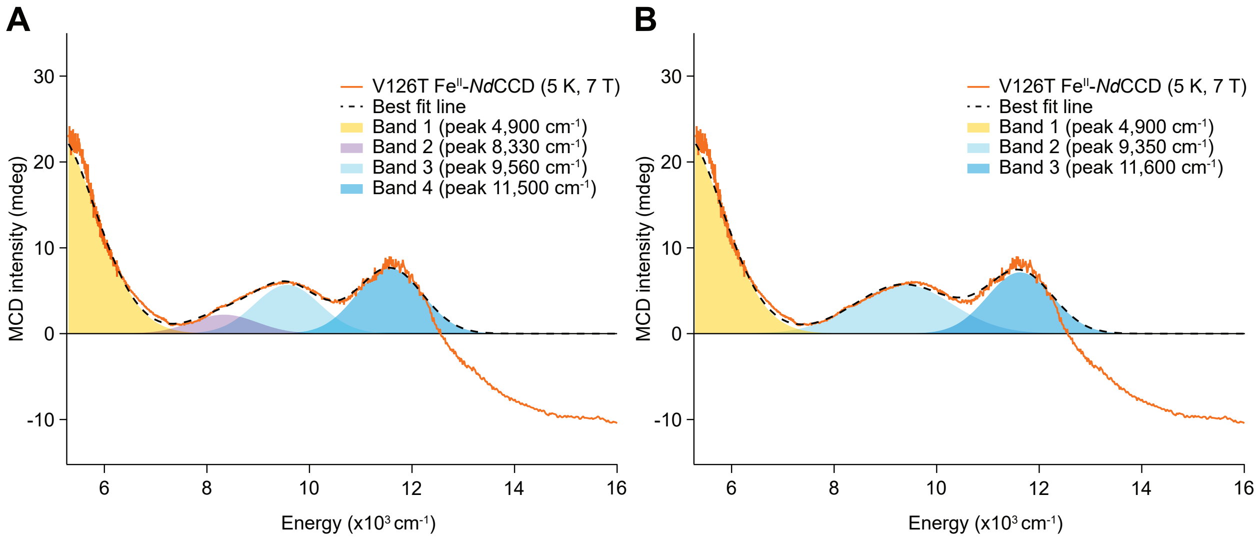

Figure S9. *Gaussian fitting of the V126T Fe^{II}-NdCCD MCD data.* **A**) Best fit of the V126T Fe^{II}-*Nd*CCD low temperature, high field (5K, 7T) MCD data. The data are shown in orange, the best fit in dashed black line, and individual fits for the features are shown in yellow (band one at 4,900 cm^{-1}), purple (band two at 8,330 cm^{-1}), light blue (band three at 9,560 cm^{-1}) and dark blue (band four at 11,500 cm^{-1}). The four features were fit with Gaussian line shapes at a full-width, half-maximum (FWHM) value of 750 cm^{-1} as found for the 6C resting site in the T151G Fe^{II}-CAO1 variant. **B**) Fit of the V126T substrate-bound, low-temperature, high-field (5K, 7T) MCD data to three Gaussian features. The data are shown in orange, the best fit as a dashed black line, and individual fits for the features are shown in yellow (band one at 4,900 cm^{-1}), light blue (band two at 9,350 cm^{-1}) and dark blue (band three at 11,600 cm^{-1}), where optimal fitting of the feature at 9,350 cm^{-1} necessitates an unrealistically large FWHM of 1,100 cm^{-1}.

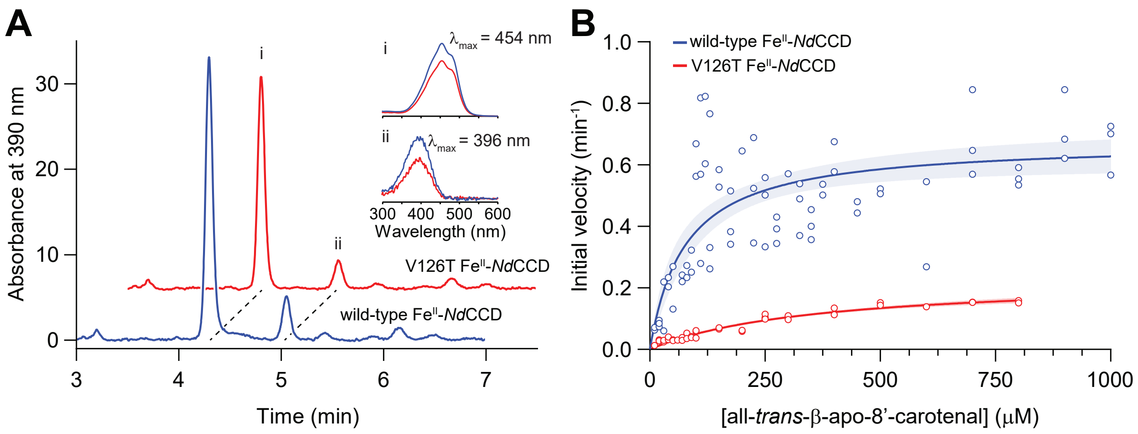

Figure S10. *All-trans-β-Apo-8'-carotenal cleavage specificity and steady-state kinetics of wild-type and V126T Fe^{II}-NdCCD.* **A**) HPLC chromatograms showing formation of all-trans-β-apo-14'-carotenal from all-*trans*-β-apo-8'-carotenal in the presence of both wild-type and V126T Fe^{II}-*Nd*CCD. The insets show absorption spectra for the substrate and product confirming their identities. **B**) Initial velocities as a function of all-*trans*-β-apo-8'-carotenal concentration are plotted for wild-type and V126T Fe^{II}-*Nd*CCD together with least-squares fits of the Michaelis-Menton equation to the data. The light-colored bands surrounding the best fit lines represent 95% confidence intervals. Best-fit Michaelis-Menton parameters are given in **Table S4**.

SUPPLEMENTARY TABLES

Table S1. Classes of oxygen-activating mononuclear non-heme Fe^{II} enzymes

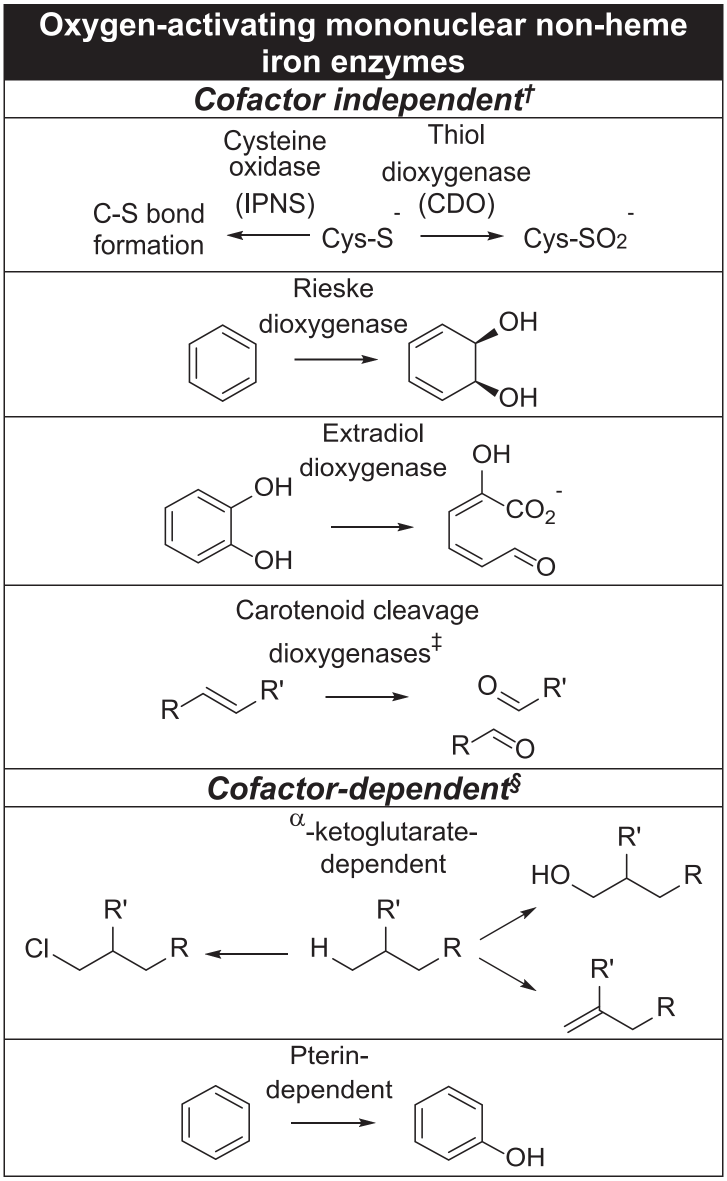

† Rieske dioxygenases require an NADH-derived electron during each catalytic cycle, which is channeled to the iron center through an iron-sulfur cluster.

‡ R and R' groups for carotenoid cleavage dioxygenase substrates contain conjugated alkene groups or aryl rings.

§ Require a two-electron-donating cosubstrate at the iron center.

Table S2. X-ray crystallographic data collection and refinement statistics†

	Wild-type Fe^{II}-CAO1	Wild-type Fe^{II}-CAO1 + PIT	T151V Fe^{II}-CAO1	T151V Fe^{II}-CAO1 + PIT	T151G Fe^{II}-CAO1	T151G Fe^{II}-CAO1 + PIT
**Data collection†**						
Beamline	NECAT ID-C	NECAT ID-E	NECAT ID-C	NECAT ID-E	NECAT ID-E	NECAT ID-E
Wavelength (Å)	1.72200	0.97918	0.97911	0.94918	0.97918	0.97918
Space group	$P3_221$	$P3_221$	$P3_221$	$P3_121$	$P3_221$	$P3_221$
Unit cell dimensions						
a, *c* (Å)	100.91, 448.82	101.19, 449.36	101.51, 450.65	100.89, 448.17	100.63, 447.66	101.31, 449.87
Resolution (Å)	50 – 2.17 (2.30-2.17)*	50-2.01 (2.13-2.01)*	50-1.90 (2.02-1.90)*	50-2.01 (2.13-2.01)*	50-2.15 (2.28-2.15)*	50-1.90 (2.02-1.90)*
R_{merge} (%)	15.2 (159.5)	23.4 (206.7)	8.8 (154.2)	15.2 (237.7)	12.4 (147.5)	11.7 (163.4)
$I / \sigma I$	17.0 (0.9)	10.5 (1.2)	11.2 (0.9)	12.8 (1.1)	9.2 (1.1)	17.5 (1.1)
Completeness (%)	89.8 (56.2)	99.8 (99.1)	99.0 (97.0)	99.9 (99.7)	99.0 (98.0)	99.9 (99.4)
Redundancy	17.4 (7.5)	6.8 (6.8)	4.0 (3.9)	10.1 (10.3)	5.1 (5.3)	9.6 (6.4)
Wilson *B*-factor (Å^2)	45	37	41	44	48	36
**Refinement**						
Resolution (Å)	50.14 – 2.17	49.36 – 2.01	48.10-1.90	50-2.01	49.10-2.15	49.99 – 1.90
No. reflections	121,762	171,471	201,172	170,141	136,427	201,407
R_{work} / R_{free} (%)	18.0/20.4	17.1/19.6	18.5/20.4	16.7/19.1	19.0/21.1	17.1/19.3
No. atoms	17,134	18,340	17,651	18,318	17,178	18,426
Protein	15,956	16,205	16,013	16,205	15,929	16,201
Iron	4	4	4	4	4	4
Water	1,143	2,023	1,630	2,001	1,235	2,023
Piceatannol	N/A	72	N/A	72	N/A	162
B-factors (Å^2)	44.1	34.5	41.2	42.5	48.5	34.8
Protein	44.0	33.5	40.8	41.4	48.5	33.7
Iron	37.7	18.8	22.1	34.5	28.3	19.6
Water	45.1	42.6	45.3	51.4	48.7	42.7
Piceatannol	N/A	29.6	N/A	39.1	N/A	48.4
R.M.S. deviations						
Bond lengths (Å)	0.003	0.004	0.005	0.004	0.003	0.005
Bond angles (°)	0.951	1.046	1.305	1.069	1.197	1.104
Ramachandran plot						
Favored (%)	97.8	97.7	98	97.4	98	97.4
Number disallowed	0	0	0	0	4‡	0
PDB accession code	5U8X§	8FU5	7T8P	8SRL	7T8Q	8FU2

† Each data set was collected from a single crystal.

* Highest-resolution shell is shown in parentheses.

‡ Pro^{263} was flagged as an outlier in each of the four subunits.

§ Re-refinement of a previously published structure [6]

Table S3. Geometrical parameters derived from CAO1 crystallographic models

	S1-metal bond (Å)	S1-metal-His angle (°)	S2-metal bond (Å)	S1-S2 interaction (Å)	S1-metal-S2 angle (°)	S1-PIT (Å)	Iron symmetry based on Procrustes distance†	PIT α-β torsion (°)
**Wild-type Fe-CAO1 (5U8X)**								
Chain A	3.04*	161.4*					SPyr (0.197)	
Chain B	2.43	162.8					SPyr (0.101)	
Chain C	2.39	161.0					SPyr (0.116)	
Chain D	2.31	158.9					SPyr (0.118)	
Average ± SD	2.38 ± 0.05	160.9 ± 1.59						
**Wild-type Fe-CAO1-PIT complex (8FU5)**								
Chain A	2.18	135.2				2.88	TBPy (0.121)	165.3
Chain B	2.16	133.2				2.85	TBPy (0.120)	165.4
Chain C	2.32	140.4				2.73	TBPy (0.143)	167.3
Chain D	2.21	152.1				2.58	SPyr (0.115)	168.7
Average ± SD	2.22 ± 0.06	140.23 ± 7.34				2.76 ± 0.12		166.68 ± 1.41
**T151V Fe-CAO1 (7T8P)**								
Chain A	2.33	157					SPyr (0.111)	
Chain B	2.28	157.9					SPyr (0.103)	
Chain C	2.41	151.3					SPyr (0.131)	
Chain D	2.44	159.1					SPyr (0.095)	
Average ± SD	2.37 ± 0.06	156.33 ± 3.00						
**T151V Fe-CAO1-PIT complex (8SRL)**								
Chain A	2.5	144.6				2.6	SPyr (0.156)	168.6
Chain B	2.3	148				2.7	SPyr (0.136)	167.9
Chain C	2.4	152.4				2.6	SPyr (0.119)	168.6
Chain D	2.3	153.5				2.7	SPyr (0.111)	170.2
Average ± SD	2.38 ± 0.08	149.63 ± 3.56				2.65 ± 0.05		168.83 ± 0.84
**T151G Fe-CAO1 (7T8Q)**								
Chain A	2.74		2.69	2.81	62		Octa (0.163)	
Chain B	2.55		2.96	2.87	62		Octa (0.167)	
Chain C	2.46		2.81	2.72	62		Octa (0.169)	
Chain D	2.34		2.74	2.75	65		Octa (0.145)	
Average ± SD	2.52 ± 0.15		2.80 ± 0.10	2.79 ± 0.06	62.75 ± 1.30			
**T151G Fe-CAO1-PIT complex (8FU2)**								
Chain A	2.21		2.14	2.46	69	2.43	Octa (0.109)	163.7
Chain B	2.02		2.31	2.25	62	2.55	Octa (0.149)	166.2
Chain C	2.19		2.39	2.37	62	2.44	Octa (0.142)	163
Chain D	2.04		2.37	2.61	72	2.49	Octa (0.114)	163.5
Average ± SD	2.12 ± 0.09		2.30 ± 0.10	2.42 ± 0.13	66.25 ± 4.38	2.48 ± 0.05		164.10 ± 1.24

* Indicates an outlier that was excluded from analysis

† The Procrustes distance, defined in [7], is given in parentheses. Lower values indicate a better match to the indicated symmetry. SPyr – Square pyramidal, TBPy – Trigonal bipyramidal, Octa – octahedral

Table S4. Wild-type and variant *Nd*CCD apparent steady-state kinetic parameters

Parameter	Wild-type *Nd*CCD	V126T *Nd*CCD
k_{cat}^{app} (s^{-1})	0.0137 ± 0.0007	0.0038 ± 0.0002
K_m^{app} (μM)	84.03 ± 20.37	362.30 ± 39.86
k_{cat}^{app}/K_m^{app} ($s^{-1} \cdot \mu M^{-1}$)	$1.63 \times 10^{-4} \pm 4.04 \times 10^{-5}$	$1.04 \times 10^{-5} \pm 1.28 \times 10^{-6}$

Values are the means ± standard errors. All-*trans*-β-apo-8’-carotenal was used as a substrate. Reactions were performed in air-saturated buffer.

Supplementary References

(1) Guindon, S.; Lethiec, F.; Duroux, P.; Gascuel, O. PHYML Online--a web server for fast maximum likelihood-based phylogenetic inference. *Nucleic Acids Res.* **2005**, *33* (Web Server issue), W557-559. DOI: 10.1093/nar/gki352

(2) Edgar, R. C. MUSCLE: multiple sequence alignment with high accuracy and high throughput. *Nucleic Acids Res.* **2004**, *32* (5), 1792-1797. DOI: 10.1093/nar/gkh340

(3) Schneider, T. D.; Stephens, R. M. Sequence logos: a new way to display consensus sequences. *Nucleic Acids Res.* **1990**, *18* (20), 6097-6100. DOI: 10.1093/nar/18.20.6097

(4) Boratyn, G. M.; Camacho, C.; Cooper, P. S.; Coulouris, G.; Fong, A.; Ma, N.; Madden, T. L.; Matten, W. T.; McGinnis, S. D.; Merezhuk, Y.; et al. BLAST: a more efficient report with usability improvements. *Nucleic Acids Res.* **2013**, *41* (Web Server issue), W29-33. DOI: 10.1093/nar/gkt282

(5) Crooks, G. E.; Hon, G.; Chandonia, J. M.; Brenner, S. E. WebLogo: a sequence logo generator. *Genome Res.* **2004**, *14* (6), 1188-1190. DOI: 10.1101/gr.849004

(6) Sui, X.; Weitz, A. C.; Farquhar, E. R.; Badiee, M.; Banerjee, S.; von Lintig, J.; Tochtrop, G. P.; Palczewski, K.; Hendrich, M. P.; Kiser, P. D. Structure and Spectroscopy of Alkene-Cleaving Dioxygenases Containing an Atypically Coordinated Non-Heme Iron Center. *Biochemistry* **2017**, *56* (22), 2836-2852. DOI: 10.1021/acs.biochem.7b00251

(7) Babai, K. H.; Long, F.; Maly, M.; Yamashita, K.; Murshudov, G. N. Improving macromolecular structure refinement with metal-coordination restraints. *Acta Crystallogr. D* **2024**, *80* (Pt 12), 821-833. DOI: 10.1107/S2059798324011458
